# Integrative Proteogenomic Characterization of Left‐Sided and Right‐Sided Colorectal Cancer

**DOI:** 10.1002/mco2.70806

**Published:** 2026-06-08

**Authors:** An Huang, Haopeng Hong, Yonghui Sun, Zhaoya Gao, Jingxuan Xu, Jiajia Chen, Yong Yang, Zhongyi Chen, Hebing Chen, Ming Li, Xiaodong Wang, Jin Gu

**Affiliations:** ^1^ Department of General Surgery, The First Affiliated Hospital, Jiangxi Medical College Nanchang University Nanchang Jiangxi China; ^2^ Department of General Surgery Peking University First Hospital Beijing China; ^3^ The Second Clinical Medical College Xinjiang Medical University Urumqi China; ^4^ Digestion and Vascular Center, Department of Pancreatic Surgery The First Affiliated Hospital of Xinjiang Medical University Urumqi China; ^5^ Department of Gastrointestinal Surgery Peking University Shougang Hospital Beijing China; ^6^ Department of Radiotherapy Peking University Shougang Hospital Beijing China; ^7^ Jingjie PTM BioLab Co. Ltd. Hangzhou China; ^8^ Academy of Military Medical Science Beijing China; ^9^ Department of Oncology Peking University Shougang Hospital Beijing China

**Keywords:** colorectal cancer, left‐sided, phosphoproteome, proteome, right‐sided, whole‐exome sequencing

## Abstract

Distinguishing characteristics have been found in the left‐sided and right‐sided colorectal cancer (CRC), which have different embryonic origins, molecular and clinical features. These result in differences in the efficacy of targeted therapy and immunotherapy. Multi‐omics characterization, predicated upon tumor laterality, may facilitate a more precise and personalized approach to the treatment of patients with CRC. Encompassing whole‐exome, proteomics, and phosphoproteomics sequencing, we conducted a comprehensive investigation of tumor and matched normal adjacent tissues from a total of 80 pairs of patients with CRC. Results revealed that the pathogenesis of left‐sided CRC was predominantly associated with chromosomal instability, while right‐sided CRC was primarily linked to microsatellite instability. Regarding the tumor microenvironment, left‐sided CRC exhibited predominant microvascular endothelial cell proliferation, while right‐sided CRC displayed enhanced MHC Class II‐associated antigen presentation mediated by M1 macrophages. Additionally, the proportion of deficient mismatch repair that developed into microsatellite instability‐high was observed to be lower in the left‐sided CRC compared to the right‐sided, indicating divergent DNA damage repair systems between laterality subtypes that contribute to differential immunotherapy efficacy. This integrated proteogenomic study provides a comprehensive and nuanced understanding of the molecular heterogeneity between left‐ and right‐sided CRC, offering opportunities for optimizing these patients' treatment outcomes through tailored therapeutic strategies.

## Graphical abstract

This study performed whole‐exome sequencing, proteomic, and phosphoproteomic analysis on 80 pairs of tumor tissues and matched normal adjacent tissues from colorectal cancer patients. The integrated proteogenomic study provides a comprehensive and nuanced understanding of the molecular heterogeneity between left‐ and right‐sided colorectal cancer, offering opportunities for optimizing these patients' treatment outcomes through tailored therapeutic strategies

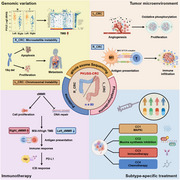

## 1 Introduction

In 2022, colorectal cancer (CRC) ranked as the third most common malignancy in incidence and the second leading cause of cancer‐related mortality worldwide, accounting for approximately 240,000 deaths in China, and 9.3% of the total cancer‐related deaths observed in China [1, 2]. Based on anatomical location and embryonic origin, CRC is categorized into right‐sided CRC (proximal to the splenic flexure of the transverse colon) and left‐sided CRC (at and distal to the splenic flexure of the transverse colon). Arising from the embryonic midgut and hindgut, respectively, right‐ and left‐sided CRC exhibit distinct clinical, genomic, and molecular profiles, increasingly recognized as separate disease entities [3].

Genomic studies have revealed that right‐sided CRC demonstrates higher frequencies of microsatellite instability‐high (MSI‐H), CpG island methylator phenotype‐high (CIMP‐H), and BRAF mutations, whereas left‐sided CRC harbors more frequent APC and TP53 mutations [4]. Furthermore, according to the consensus molecular subtypes (CMS), left‐sided CRC is predominantly CMS2 and CMS4, while right‐sided CRC is enriched for CMS1 and CMS3. These molecular distinctions underpin divergent prognoses [5]. In metastatic CRC (mCRC), primary tumor location influences responses to palliative chemotherapy, targeted therapy, and immunotherapy. Combining systemic chemotherapy with bevacizumab reduces mortality in both right‐ and left‐sided mCRC compared to chemotherapy alone, while cetuximab combined with chemotherapy correlates with reduced mortality exclusively in left‐sided mCRC [6]. The NCCN guidelines now incorporate tumor laterality into treatment considerations for unresectable mCRC, with anti‐EGFR therapy recommended only for RAS wild‐type, left‐sided CRC [7]. Subgroup analysis of the KEYNOTE‐177 trial further demonstrated that pembrolizumab significantly improved progression‐free survival (PFS) compared to traditional chemotherapy in right‐sided deficient mismatch repair (dMMR)/MSI‐H mCRC, which was not observed in left‐sided cases [8]. Elucidating the mechanisms driving these disparities is critical for patient stratification and personalized therapeutic strategies.

Current clinical decision‐making for CRC prioritizes TNM staging, DNA mismatch repair status, and RAS/BRAF mutation profiling. Although proteogenomic studies have identified novel biomarkers and therapeutic targets [9], the proteogenomic landscape of CRC has yet to be systematically characterized in large cohorts stratified by tumor laterality. Multi‐omics characterization grounded in tumor parochialism holds the potential to enhance the precision and personalization of diagnosis and treatment.

To address this gap and enable more precise and individualized therapies, we conducted integrated genomic, proteomic, and phosphoproteomic analyses on 80 paired Chinese CRC specimens following guidelines established by the Clinical Proteomic Tumor Analysis Consortium (CPTAC) [10]. This comprehensive approach systematically delineates the proteogenomic features distinguishing left‐ and right‐sided CRC. The pathogenesis of left‐sided CRC was predominantly associated with chromosomal instability, while right‐sided CRC was primarily linked to microsatellite instability. Furthermore, the left‐sided and right‐sided CRC inhabited distinct tumor microenvironments and possessed divergent DNA damage repair systems, which contributed to variances in immunotherapy efficacy. Conclusive evidence demonstrated that molecular subtypes could effectively identify patients' biologic characteristics and therapeutic sensitivity.

## 2 Results

### 2.1 Proteogenomic Molecular Profiling of Chinese Left‐Sided and Right‐Sided CRC

**FIGURE 1:**
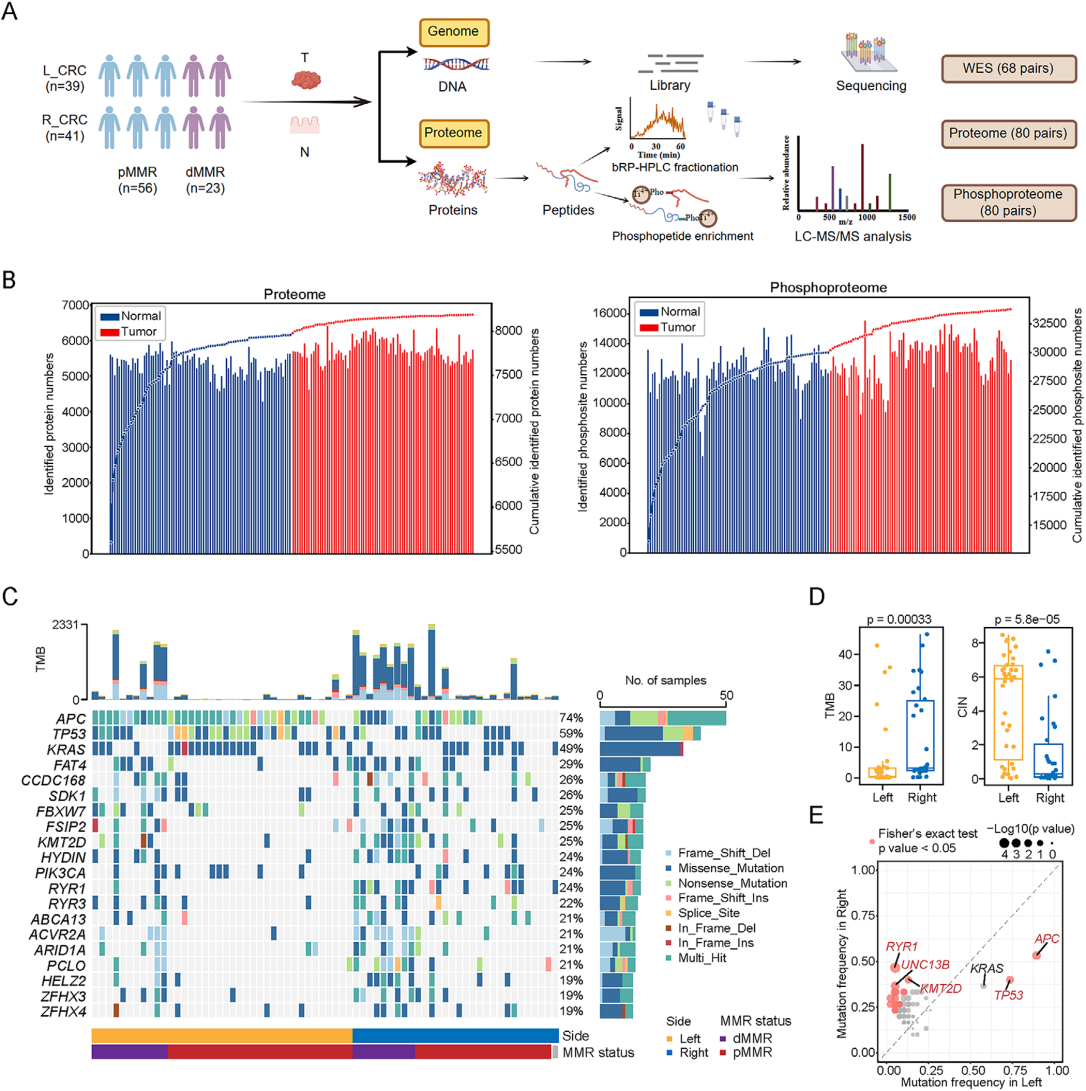
Proteogenomic molecular profiling of Chinese left‐sided and right‐sided CRC. (A) Overview of the experimental design. (B) Overview of proteomic and phosphoproteomic profiles of CRC samples. Number of proteins and phosphorylation sites identified per sample. (C) Genomic profile of the CRC samples with WES data. Left side, mutation of the top 20 genes in each sample. Right side mutation types and their frequencies. (D) Comparisons of TMB and CIN between left‐sided and right‐sided CRC in this cohort. Right‐sided CRC (*n* = 30) haa significantly higher TMB and lower CIN than left‐sided CRC (*n* = 38, *p*‐values are derived from the Wilcoxon rank‐sum test). (E) Comparisons of gene mutation frequencies between left‐sided and right‐sided CRC. The frequency of mutations in *APC* and *TP53* in the classical oncogenic pathway of the right‐sided CRC is significantly higher than that of the left right‐sided (*p*‐values are derived from two‐sided Fisher's exact test).

To characterize the proteogenomic landscape of left‐ and right‐sided CRC in the Chinese population, we retrospectively collected 80 pairs of treatment‐naïve primary CRC tumor tissues and matched normal adjacent tissues (NATs) from surgical resections under standardized protocols (Peking University Shougang Hospital [PKUSG]‐CRC cohort). To account for potential confounding effects beyond tumor location and MMR status, we implemented a matching strategy. Following matching, key clinicopathological features, such as age, sex distribution, and tumor stage, demonstrated no significant differences between left‐sided and right‐sided CRC (Table S1). The schematic of the experimental design is depicted in Figure 1A. In sum, whole‐exome sequencing (WES) identified a total of 29,129 non‐silent somatic mutations, 96,806 silent mutations, and 17 significant arm‐level somatic copy number alteration (SCNA) events (*q* < 0.05). Proteome quantified 8193 proteins, and phosphoproteome detected 33,796 high‐confidence phosphosites across 6815 phosphorylated proteins (Figure 1B). QC analyses revealed high consistency and stability of the MS platforms, with median correlation coefficients among QC replicates of 0.973 (proteome) and 0.98 (phosphoproteome) (Figure S1A,B). Tumor tissues exhibited significantly higher protein and phosphosite identification rates compared to NATs (Figure S1C–E). Inter‐sample correlations for tumor tissues ranged from 0.745 to 1.0 (median 0.863), while NATs showed correlations between 0.678 and 1.0 (median 0.905), reflecting tumor tissue heterogeneity (Figure S1F).

### 2.2 Somatic Alterations and Mutational Profiles of Chinese Left‐Sided and Right‐Sided CRC

Within the PKUSG‐CRC cohort, the most frequently mutated genes were APC (74%), TP53 (59%), and KRAS (49%) (Figure 1C). The classical adenoma‐carcinoma sequence in CRC pathogenesis diverges into two major pathways: chromosomal instability (CIN) and MSI. Comparative analysis of tumor mutation burden (TMB) and CIN between left‐ and right‐sided CRC revealed a median TMB of 5.06 non‐synonymous mutations per megabase (mut/Mb) in left‐sided CRC, significantly lower than that of right‐sided CRC (13.00 mut/Mb) (Figure 1D). Conversely, left‐sided CRC exhibited markedly higher CIN, supporting divergent oncogenic mechanisms. Further mutation frequency analysis demonstrated that APC and TP53 alterations were enriched in left‐sided CRC, consistent with predominant CIN‐driven tumorigenesis (Figure 1E).

**FIGURE 2:**
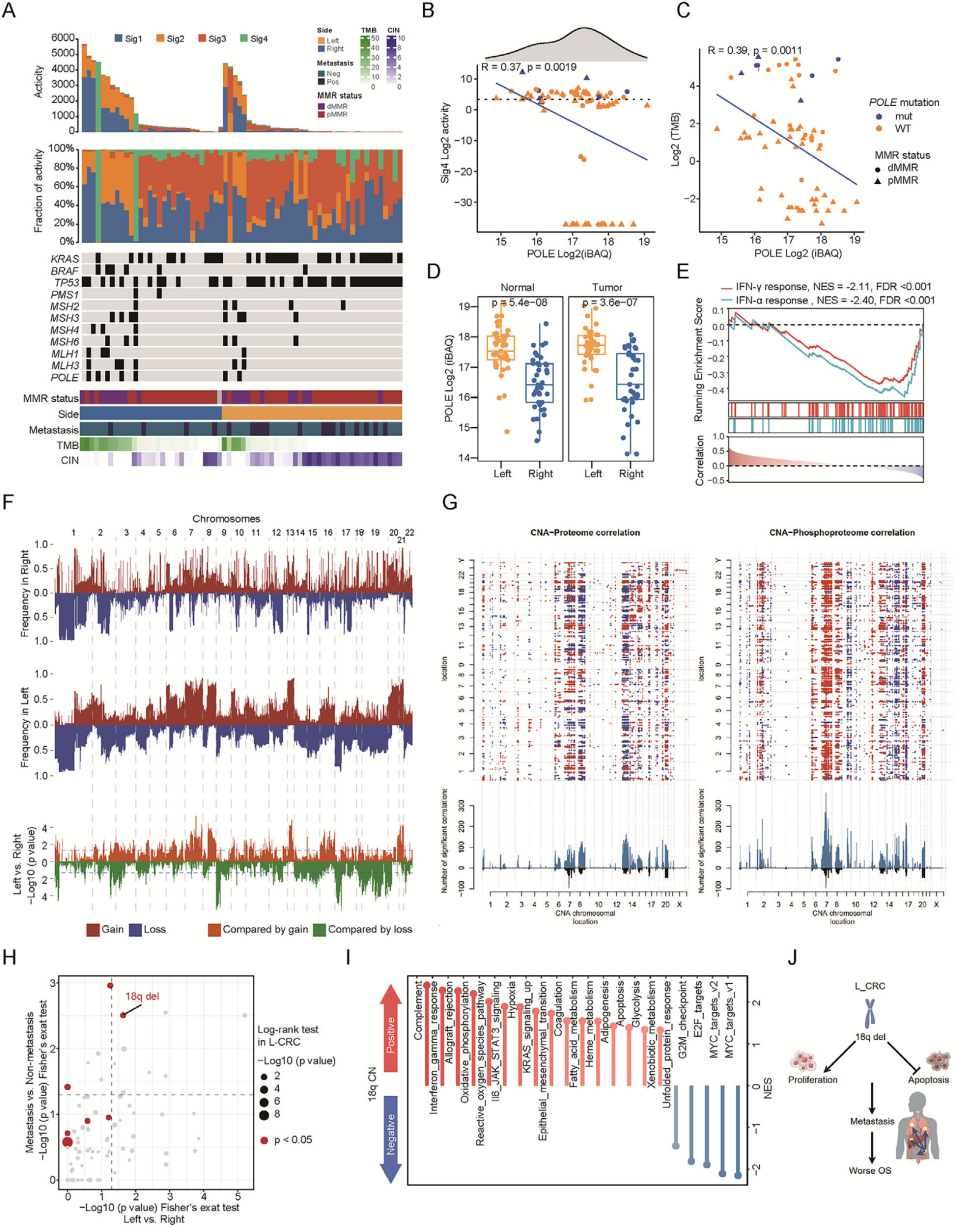
Somatic alterations and mutational profiles of Chinese left‐sided and right‐sided CRC. (A) Mutation signature activities detected in this study. Mutations including *KRAS*, *BRAF*, *TP53*, mismatch repair genes, and *POLE* are also shown. (B) Scatter plot showing the correlation between POLE expression levels and Sig4 activities (Spearman's correlation). POLE protein expression negatively correlates with Sig4 activity. (C) Scatter plot showing the correlation between POLE expression levels and TMB (Spearman's correlation). POLE protein expression negatively correlates with TMB. (D) Comparison of POLE between Left‐CRC and Right‐CRC in tumor and adjacent tissues. In both tumor and NATs, the expression of POLE protein is lower in the right‐sided (*n* = 41) than in the left‐sided CRC (*n* = 39, *p*‐values are derived from the Wilcoxon rank‐sum test). (E) GSEA plot showing that interferon response pathways (Hallmarks) negatively correlate with POLE expression levels. (F) Comparison of gene‐level CNA events between Left‐CRC and Right‐CRC in this cohort. The upper plot illustrates the frequency of CNA events, and the lower plot illustrates the −log 10 (*p*‐value) of each gene for the comparison of Left‐CRC and Right‐CRC (*p*‐value from two‐sided Fisher's exact test). (G) Effect of CNA on proteins and phosphorylated proteins. Top: correlations of CNA (*x* axes) with protein (left panel) and phosphoprotein (right panel) abundance (*y* axes). Significant (FDR <0.05) positive (red) and negative (blue) correlations are shown. Diagonal lines indicate cis effects of CNA on mRNA or protein. Bottom: the number of proteins or phosphorylated proteins significantly associated with a specific CNA. Blue bars: correlations specific to proteins or phosphorylated proteins. Black bars: CNAs associated with both proteins and phosphorylated proteins. (H) CNA events associated with tumor laterality and metastasis. The 18q deletion is the only arm‐level CNA event correlating with Left‐CRC metastasis, prompting poor prognosis (*p*‐value from two‐sided Fisher's exact test). (I) GSEA analysis of the 18q deletion cis and trans effects on Hallmarks. Red indicates pathways positively associated with 18q CN, and blue indicates pathways negatively associated with 18q CN. (J) Schematic representation of 18q deletion leading to poor prognosis in Left‐CRC.

Non‐negative matrix factorization (NMF) of the mutational landscape delineated four mutational signatures (Figure 2A and Figure S2A). Signature 2 (Sig2) correlated with dMMR, while Signature 4 (Sig4) was linked to POLE exonuclease domain mutations. POLE mutant (POLE^mut^) exhibited significantly stronger Sig4 activity compared to POLE wild‐type (POLE^wt^) (Figure 2A and Figure S2B). Present studies demonstrate that POLE^mut^ elicits a substantial benefit in immunotherapy, but it occurs in only 1%–2% of CRC patients. We observed that decreased POLE expression phenocopied POLE^mut^ ‐associated effects. Specifically, POLE expression inversely correlated with Sig4 activity (Spearman *R* = 0.37, *p* = 0.0019, Figure 2B) and TMB (*R* = 0.39, *p* = 0.0011, Figure 2C). Notably, POLE expression was significantly reduced in right‐sided CRC tumors compared to left‐sided counterparts. This phenomenon was observed not only in tumor tissues but also in NATs (Figure 2D), indicating that the origins of the left‐sided and right‐sided CRC might be distinct. GSEA further revealed that low POLE expression inversely correlated with IFN‐α and IFN‐γ signaling pathways (Figure 2E), implicating heightened immune activation in right‐sided CRC.

WES‐based SCNA profiling identified 25 chromosome‐level SCNA events in left‐sided CRC, alongside 6336 gene‐level gains (26.10%) and 7600 gene‐level losses (31.31%) compared to right‐sided CRC (Figure 2F and Figure S2C). Analysis of CNA‐driven proteomic and phosphoproteomic regulation identified 260,271 CNA–protein pairs and 350,700 CNA–phosphoprotein pairs with significant correlations (FDR <0.05). Cis/Trans regulatory hotspots localized to chromosomes 7, 8, 13, 15, and 20 (Figure 2G). Metastasis‐specific comparisons highlighted 2p loss, 15q loss, and 18q loss as potential mediators of metastasis in left‐sided CRC (Figure 2H). Survival analysis associated 18q deletion (18q del) with poor overall survival (OS) in left‐sided CRC (log‐rank test, *p* = 0.02, Figure 2H and Figure S2D). Functional annotation revealed that 18q‐associated proteins inversely correlated with G2/M checkpoint, E2F, and MYC‐driven proliferation pathways, while positively correlating with apoptosis (Figure 2I). These findings suggest that 18q del may drive metastasis in left‐sided CRC by suppressing apoptosis and enhancing proliferative capacity, thereby worsening prognosis (Figure 2J).

### 2.3 Proteomics and Phosphoproteomics Features in CRC Tumor Tissues Compared With NATs

**FIGURE 3:**
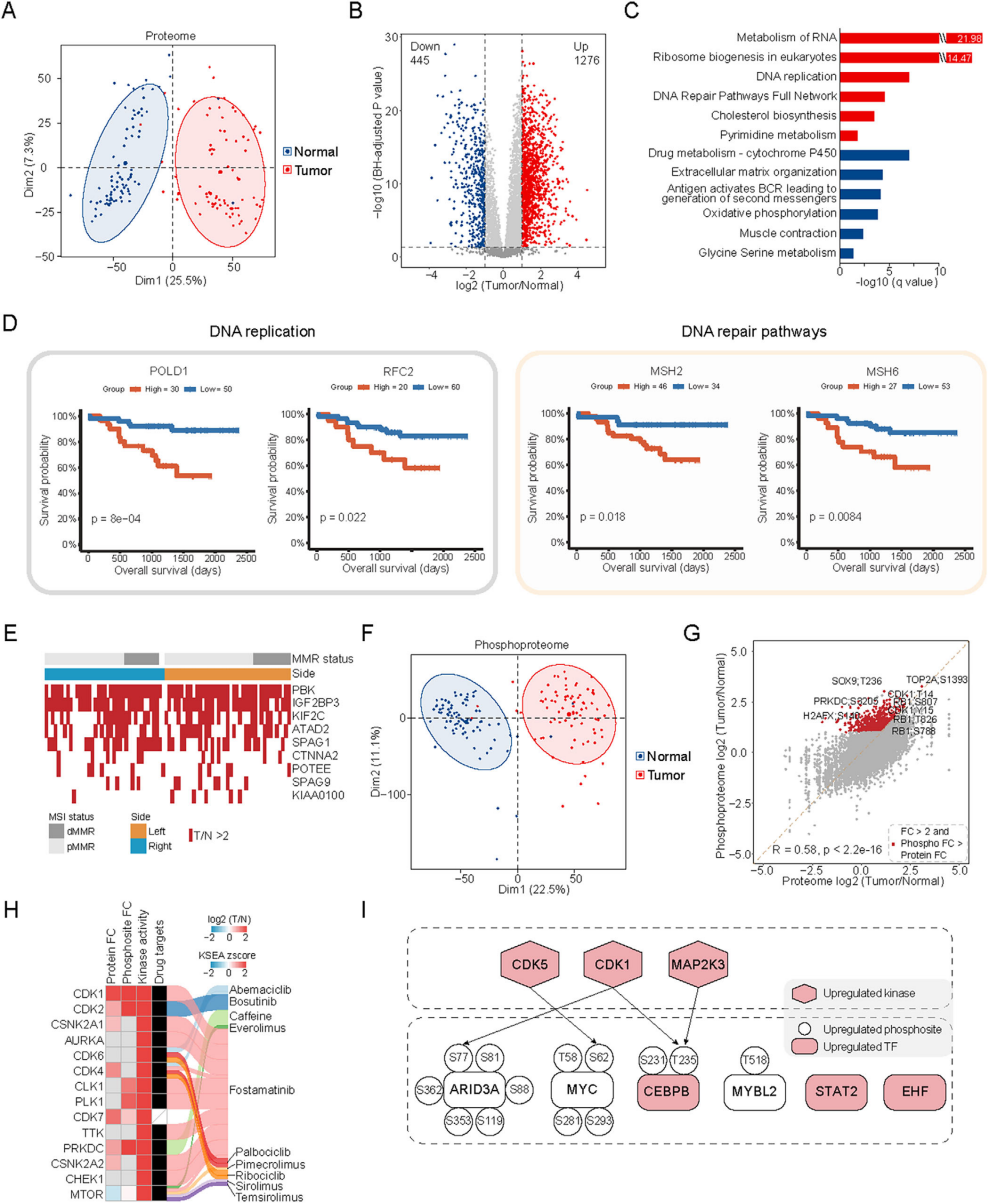
Proteomics and phosphoproteomics landscape of CRC. (A) PCA of proteome data in 80 paired tumor and adjacent tissue samples. (B) Volcano plot showing DEPs (two‐sided paired *t*‐test, Benjamini–Hochberg‐adjusted *p*‐value <0.05, FC >2) in tumor and adjacent tissues. Proteins that were significantly overexpressed in tumor/adjacent tissues are presented with red/blue‐filled scatters. (C) Pathway overrepresented analysis of tumor/adjacent DEPs using ConsensusPathDB. (D) Overexpression of proteins such as POLD1 and RFC2 in the DNA replication pathway, and MSH2 and MSH6 in the DNA damage repair pathway, is associated with poor prognosis in CRC (*p*‐values derived from the log‐rank test). (E) Nine CT antigens overexpressed by at least two‐fold in tumors compared to NATs in more than 5% of all samples. (F) PCA of phosphoproteome data in 80 paired tumor and adjacent tissue samples. (G) Comparison of abundance changes between phosphosites and their corresponding proteins. A total of 1357 phosphorylation sites are found to be more than twice as abundant in tumors as in NATs, and the change in phosphorylation site abundance exceeds the corresponding change in protein abundance. (H) Heatmap illustrating kinases of increased activity inferred by KSEA or increased protein and phosphorylation levels. Gray boxes indicate the data are not available. Black boxes indicate the presence of an FDA‐approved drug or a drug in clinical trials for the kinase. Kinase‐drug information is from drugbank. Kinases with FDR less than 0.05 are considered significant. (I) Increased TF substrate activities and kinases with TF substrates. The red diamonds represent kinases upregulated in CRC that regulate the expression of TFs (red squares) through a number of phosphorylation sites (white circles).

Among the 8193 proteins identified via LC‐MS/MS, 6095 were detected in more than 50% of tumors or NATs and were retained for downstream analyses. PCA demonstrated a distinct demarcation between tumor and NATs (Figure 3A). Differential expression analysis revealed 1721 differentially expressed proteins (DEPs; Figure 3B), with 1276 upregulated and 445 downregulated in tumors. ConsensusPathDB‐based molecular interaction analysis highlighted upregulated protein enrichment in RNA metabolism, eukaryotic ribosome biogenesis, DNA replication, and DNA repair pathways (FDR <0.05), whereas downregulated proteins were associated with cytochrome P450‐mediated drug metabolism, extracellular matrix organization, and oxidative phosphorylation (Figure 3C). Notably, proteins involved in DNA replication or damage repair, such as POLD1, RFC2, MSH2, and MSH6, correlated with patient prognosis (Figure 3D).

We identified nine candidate cancer‐testis (CT) antigens (Figure 3E) with potential as vaccine targets due to their immunogenicity in cancer patients. Survival analysis revealed poor prognosis for patients with high expression of ATAD2, CTNNA2, or KIF2C, whereas elevated SPAG9 was associated with favorable outcomes (Figure S3A). For biomarker discovery, 94 high‐confidence biomarkers were identified after thorough selection (Figure S3B), 14 of which overlapped with the 31 CRC biomarkers previously reported by Vasaikar et al. (Figure S3C,D).

Comparative phosphoproteomic profiling revealed profound disparities in phosphorylation site activity between tumors and NATs (Figure 3F). Among 1528 tumor‐upregulated phosphorylation sites (log2 fold‐change >1, Figure S3E), 1357 (81%) exhibited that phosphorylation‐level changes exceeded corresponding protein abundance shifts (Figure 3G). Integration of proteomic and phosphoproteomic data demonstrated significant concordance between upregulated proteins and their phosphorylation sites (Spearman *R* = 0.58). We further identified 729 CRC‐activated phosphosites, including 85 tumor‐associated sites (e.g., RB1, CDK1, SOX9). Notably, hyperphosphorylation of H2AFX S140 (a biomarker of DNA double‐strand breaks) was significantly enriched in left‐sided CRC (Figure S3F), suggesting a mechanistic link to CIN‐associated tumorigenesis. Kinase‐substrate network analysis suggested that PRKDC activity correlated most strongly with H2AFX S140 phosphorylation, implicating PRKDC as the most probable regulator of H2AFX S140 phosphorylation (Figure S3G).

KSEA of the phosphoproteome identified kinases with differential activity between tumor and NATs, including CDK1, CDK2, and CSNK2A1 (Figure S3H,I). Comprehensive integration of protein expression, phosphosite modifications, kinase activity, and drug targets nominated fostamatinib (a SYK inhibitor) and ribociclib (a CDK4/6 inhibitor) as candidate therapeutic agents (Figure 3H). Further interrogation of TF activity using VIPER predicted four downregulated (IRF4, TCF4, IKZF1, STAT3) and six upregulated TFs (CEBPB, EHF, ARID3A, MYBL2, STAT2, MYC), corroborated by protein‐ or phospho‐level evidence. Network analysis linked tumor‐upregulated kinases CDK1, CDK5, and MAP2K3 to phosphorylated TFs, enabling construction of a TF‐kinase regulatory network central to CRC progression (Figure 3I).

### 2.4 Exploring Differences Between Left‐ and Right‐Sided CRC From Proteome and Phosphoproteome

**FIGURE 4:**
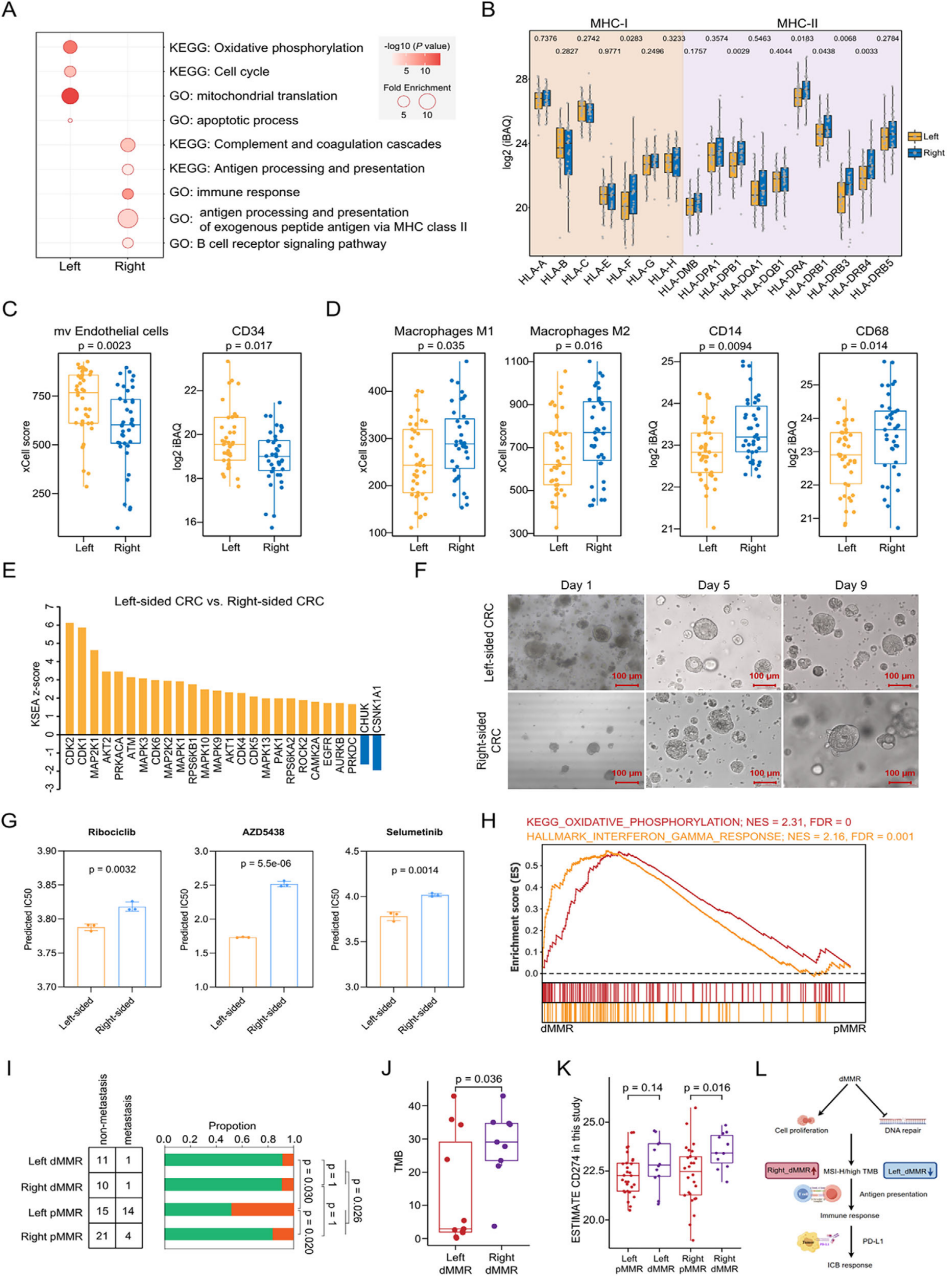
Differences in proteomics and phosphoproteomics between right‐sided and left‐sided CRC. (A) Pathway overrepresents analysis of right‐sided/left‐sided CRC differentially expressed proteins. (B) Comparison of MHC‐I and MHC‐II proteins between right‐sided (*n* = 41) and left‐sided CRC (*n* = 39) tumor samples. *p*‐values are derived from the Wilcoxon rank‐sum test. (C) Boxplots showing the difference of microvascular endothelial cells and vascular endothelial marker CD34 between right‐sided (*n* = 41) and left‐sided CRC (*n* = 39, Wilcoxon rank‐sum test). (D) Boxplots showing the difference of macrophage M1 and M2 and macrophage markers CD14 and CD68 between right‐sided (*n* = 41) and left‐sided CRC (*n* = 39, Wilcoxon rank‐sum test). (E) Differential analysis of kinase activities by right‐sided and left‐sided CRC. Orange: kinases with increased activity in left‐sided CRC. Blue: kinases with increased activity in right‐sided CRC. (F) Morphology of right‐sided and left‐sided CRC organoids on the first, fifth, and ninth days. (G) The sensitivity of right‐sided (*n* = 3) and left‐sided CRC (*n* = 3) organoids to ribociclib, AZD5438, and selumetinib based on GDSC2 (two‐sided unpaired *t*‐test). (H) GSEA plot showing that oxidative phosphorylation (KEGG gene set) and interferon gamma response (Hallmark gene set) are upregulated in dMMR tumors. (I) Barplots showing the distribution of metastasis and nonmetastasis tumors among tumors with distinct MMR status in right‐sided and left‐sided CRC tumors (Fisher's exact test). Green: nonmetastasis. Orange: metastasis. (J) Comparison of TMB between right‐sided (*n* = 9) and left‐sided dMMR CRC (*n* = 9) tumors (two‐sided unpaired *t*‐test). (K) Comparison of estimated CD274 levels between dMMR (*n* = 23) and pMMR (*n* = 56) tumors in both right‐sided (*n* = 40) and left‐sided CRC (*n* = 39, two‐sided unpaired *t*‐test). (L) Schematic representation of the differential benefit of immune therapy between right‐sided and left‐sided dMMR CRC tumors.

DEP analysis between left‐ and right‐sided CRC identified 44 upregulated proteins in left‐sided CRC and 69 in right‐sided CRC. KEGG and GO enrichment analyses revealed distinct functional polarization: left‐sided CRC exhibited pathway enrichment in oxidative phosphorylation, cell cycle regulation, mitochondrial translation, and apoptosis, whereas right‐sided CRC was markedly enriched in immune response and antigen presentation pathways (Figure 4A). Strikingly, MHC‐II molecules, including HLA‐DPB1, HLA‐DRA, and HLA‐DRB3, but not MHC‐I, were overexpressed in right‐sided CRC (Figure 4B).

xCell‐based immune infiltration evaluation demonstrated higher microvascular endothelial cell infiltration in left‐sided CRC (Wilcoxon rank‐sum test, *p* = 0.0023; Figure 4C), corroborated by elevated expression of CD34, a microvascular endothelial cell marker. Microvascular endothelial infiltration positively correlated with cell cycle activity (Spearman *p* = 0.37, *p* = 0.00062; Figure S4A), implicating angiogenic support for tumor proliferation. Conversely, right‐sided CRC harbored elevated M1 and M2 macrophage infiltration, supported by overexpression of macrophage markers CD14 and CD68 (Figure 4D). Antigen presentation pathway enrichment in right‐sided CRC was predominantly driven by M1 macrophage activity (Spearman *p* = 0.36, *p* = 0.0011; Figure S4B), aligning with MHC‐II protein enrichment. Through multiplex immunofluorescence analysis, we observed significantly enriched CD34^+^ cells infiltration in left‐sided CRC, whereas CD68^+^and CD163^+^ macrophage populations were more abundant in right‐sided CRC (Figure S4C). Kinase activity profiling revealed hyperactivation of CDK, MAPK, AKT, and EGFR families in left‐sided CRC (Figure 4E), likely underpinning the superior clinical response to cetuximab in left‐sided CRC. In our preliminary study involving organoid construction and RNA‐seq analysis of CRC tumor samples, when grouped based on tumor location for drug sensitivity analysis, left‑sided CRC exhibited greater sensitivity to the CDK inhibitors ribociclib and AZD5438, as well as to the MAPK pathway inhibitor selumetinib (Figure 4F,G). These findings further support the potential utility of CDK inhibitors in left‐sided CRC.

Subsequently, proteomic profiling confirmed diminished expression of MMR proteins, such as MSH2, MSH3, and MSH6, in dMMR versus proficient MMR (pMMR) CRC, while RPL22L1 (an MSI‐associated protein) was overexpressed in dMMR patients (Figure S4D), validating dataset integrity. GSEA revealed significant upregulation of oxidative phosphorylation and IFN‐γ signaling pathways in dMMR CRC (Figure 4H). Single‐cell RNA‐seq analysis of dMMR CRC patients revealed elevated angiogenesis scores in left‐sided CRC, whereas antigen processing and presentation scores were heightened in right‐sided CRC. Further dissection of conventional dendritic cells (cDCs) demonstrated predominant enrichment of CLEC9A^+^ cDCs in right‐sided CRC (Figure S4E–J). Stratifying by tumor laterality, left‐sided dMMR CRC preferentially activated oxidative phosphorylation, whereas right‐sided dMMR CRC was primarily associated with immune activation, for example, the interferon pathway (Figure S4K,L).

Furthermore, pMMR CRC demonstrated elevated metastatic risk compared to dMMR CRC in left‐sided CRC, while it was absent in the right‐sided CRC (Figure 4I). Mirroring the KEYNOTE‐177 subgroup analysis, where pembrolizumab improved PFS in right‐sided dMMR/MSI‐H CRC but not in left‐sided mCRC, we investigated underlying mechanisms. Notably, right‐sided dMMR CRC exhibited higher TMB versus left‐sided dMMR CRC (two‐sided unpaired *t*‐test, *p* = 0.036; Figure 4J). Validation in the SYSUCC CRC cohort observed a similar phenomenon (Figure S4M) and revealed MSI‐H prevalence of 74.58% in right‐sided dMMR CRC versus 34.48% in left‐sided dMMR CRC (Fisher's exact test, *p* = 0.002; Figure S4N). TCGA CRC cohort further linked right‐sided dMMR to CD274 (PD‐L1) overexpression (Figure S4O), and no such pattern existed in the left‐sided dMMR CRC. As CD274 was undetected in our proteomic dataset, we developed a prediction model using the CPTAC and TCGA CRC cohorts. The Pearson's correlation between true CD274 and estimated CD274 levels was 0.86 in the CPTAC and 0.83 in the TCGA CRC cohort (Figure S4P). Applied to the PKUSG‐CRC cohort, this model confirmed higher CD274 expression in right‐sided dMMR CRC than pMMR (two‐sided unpaired *t*‐test, *p* = 0.016; Figure 4K), which was absent in the left‐sided patients. Collectively, dMMR drives TMB/MSI‐H phenotypes via promoting cell proliferation and inhibiting DNA damage repair, enabling neoantigen presentation and immune activation. However, this axis is attenuated in left‐sided dMMR CRC, likely explaining their reduced immunotherapy responsiveness (Figure 4L).

### 2.5 Proteomic Subtypes of CRC in the Chinese Population

**FIGURE 5:**
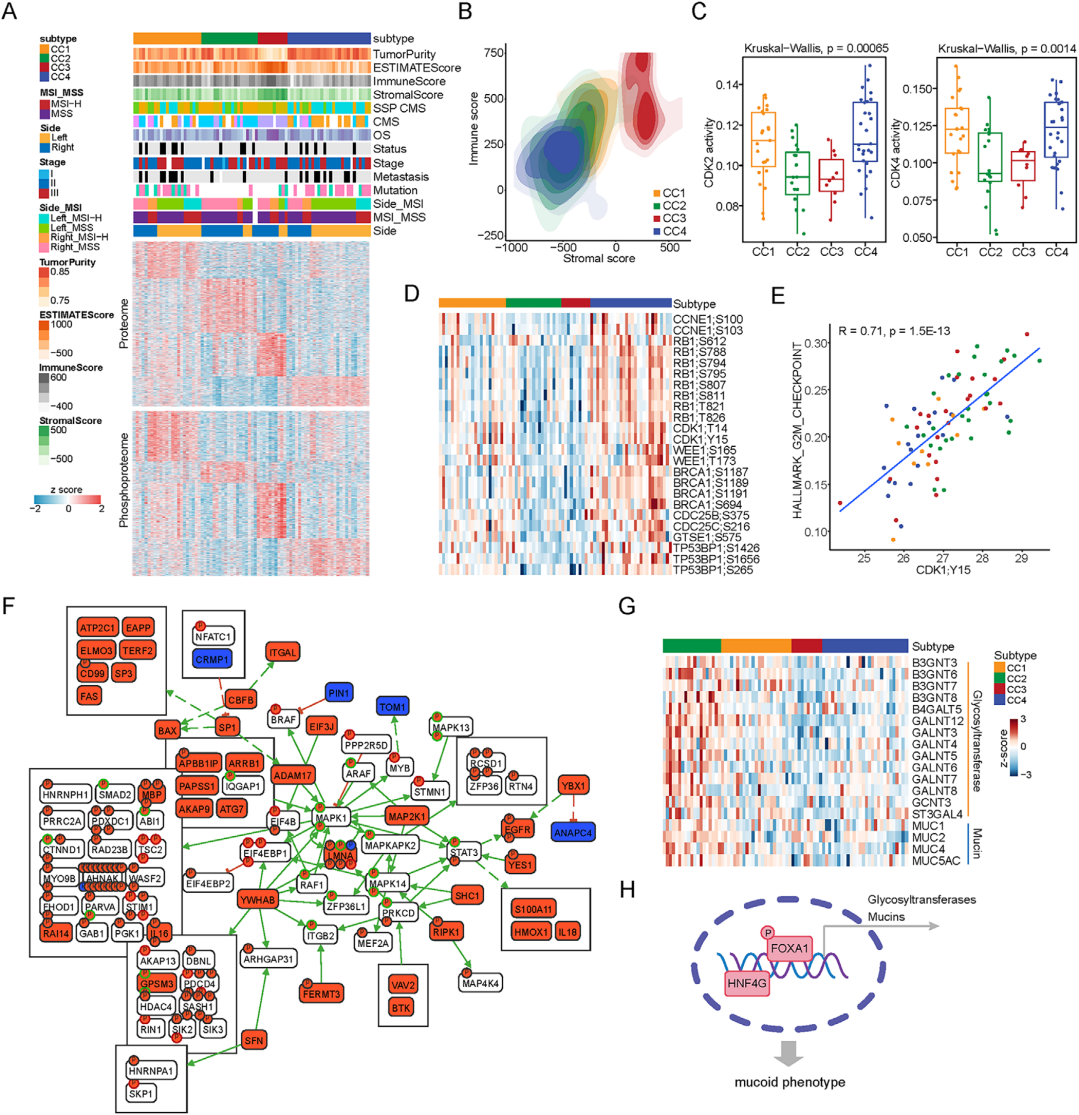
Proteomics‐based construction of molecular subtypes and their characteristics. (A) Relative abundances of upregulated proteins and phosphosites in the four proteomic subtypes. Associations of proteomic subtypes with clinical and molecular features are also shown. (B) Two‐dimensional density plot for CRC proteomic subtypes based on ESTIMATE immune score and stromal score. (C) Boxplots showing the differences of CDK2 activities and CDK4 activities among subtypes (Kruskal–Wallis test). CC1: *n* = 23. CC2: *n* = 19. CC3: *n* = 10. CC4: *n* = 28. (D) Heatmap showing the differential phosphosites involved in cell cycle regulation among proteomic subtypes. (E) Correlation analysis between CDK1 Y15 phosphorylation abundances and Hallmark G2M checkpoint scores (Spearman's correlation). (F) Pathway and causality analysis using CausalPath. The protein–protein network diagram showing proteins and phosphosrylations upregulated or downregulated in CC1 compared with other subtypes. (G) Heatmap showing the differential protein abundances involved in mucin type O‐glycan biosynthesis among proteomic subtypes. Multiple glycosyltransferases and mucins are generally highly expressed in the CC2 subtype. (H) Schematic representation of HNF4G and FOXA1‐mediated upregulation of mucin type O‐glycan biosynthesis in CC2 tumors.

Unsupervised clustering of proteomic data from 80 CRC patients identified four molecularly distinct subtypes (Clusters CC1–CC4; Figure 5A and Figure S5A–C). These subtypes exhibited divergent molecular and clinical profiles. CC1 and CC4 were predominantly left‐sided CRC with higher metastatic prevalence (Figure S5D), whereas CC2 and CC3 were enriched in right‐sided. GSEA revealed marked activation of proliferation‐related pathways (MYC, E2F, G2/M checkpoint) in CC4, while CC3 showed robust immune pathway enrichment, such as IFN‐γ, TNF‐α, and complement (Figure S5E). Consistent with this, ESTIMATE score further indicated that CC3 subtype exhibited the highest degree of immune infiltration, aligning with a “hot tumor” phenotype (Figure 5B and Figure S5F). Phosphoproteomic profiling further highlighted elevated activity of kinases CDK2 and CDK4 in CC4 (Figure 5C), alongside hyperphosphorylation of key proliferation regulators (RB1, CDK1, TP53BP1) at multiple residues in CC1 and CC4 (Figure 5D). CDK1 Y15 phosphorylation correlated strongly with G2/M checkpoint regulation pathway activity (Spearman *p* = 0.71, *p* = 1.5E‐13; Figure 5E). Similarly, RB1 phosphorylation correlated with E2F signaling (Figure S5G), confirming CC4 as a hyperproliferative subtype.

**FIGURE 6:**
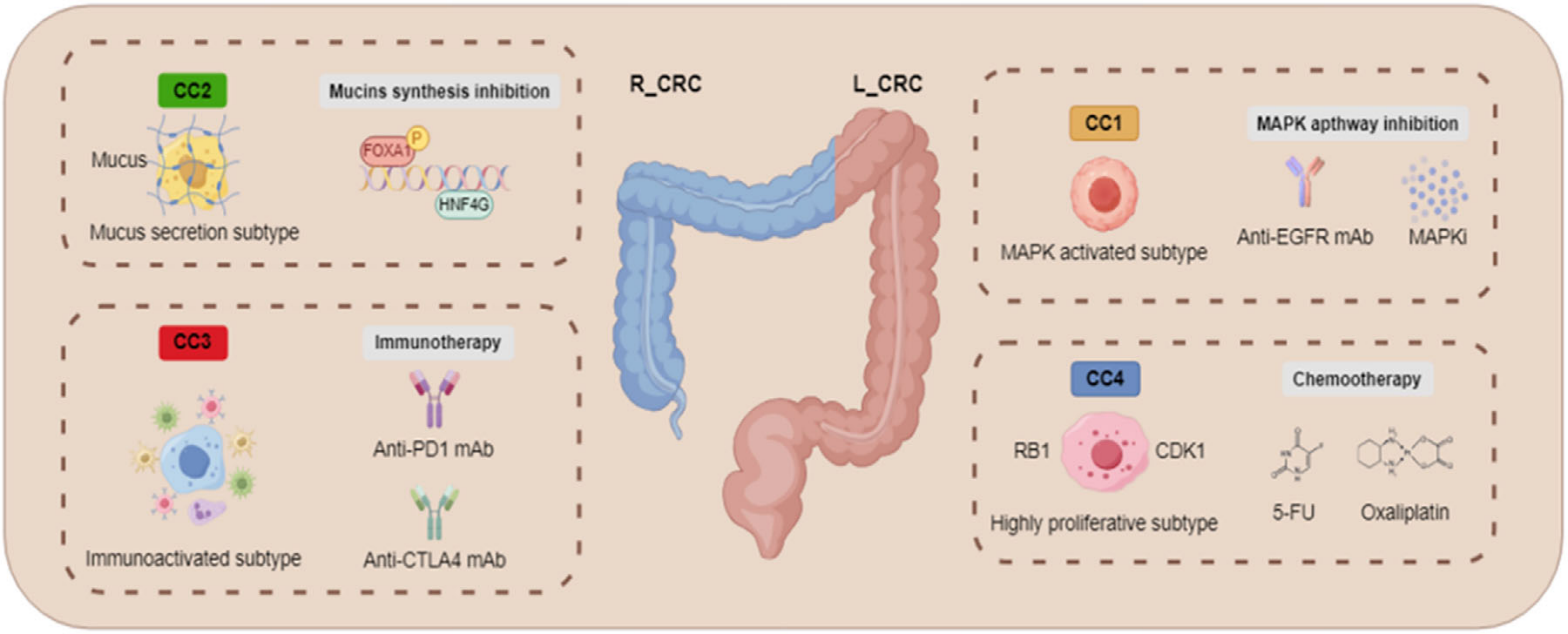
Summary of four proteomic subtypes.

CausalPath analysis of CC1 for kinase/phosphatase substrates as well as TFs identified MAPK pathway dominance, with multiple MAPK family kinases activated (Figure 5F). And multiple MAPK family proteins, such as MAPK1, MAP2K1, and MAP2K2, were upregulated in CC1, indicating that CC1 is a MAPK‐activated subtype. In CC2, KEGG enrichment revealed a significant activation of the O‐glycan biosynthetic pathway, supported by overexpression of glycosyltransferases and mucins (e.g., MUC2, MUC5AC; Figure 5G). In combination with the proteomic and phosphoproteomic data, TF regulator analysis prioritized FOXA1 and HNF4G, which were uniquely phosphorylated/activated in CC2 (Figure S5J). We propose that FOXA1 phosphorylation and HNF4G activation drive glycosyltransferase and mucin upregulation, underpinning CC2's mucus‐secretion phenotype (Figure 5H). A consolidated molecular profile of the four proteomic subtypes delineates their distinct biological and clinical attributes (Figure 6).

## 3 Discussion

Left‐ and right‐sided CRC exhibit distinct embryological origins, epigenomic landscapes, genomic alterations, transcriptomic profiles, proteomic signatures, and microbiome compositions [4, 11, 12, 13, 14], underpinning differential prognostic outcomes, responses to anti‐EGFR monoclonal antibodies, and immunotherapeutic sensitivities. In this study, we present the first integrative multi‐omics characterization (genome, proteome, phosphoproteome), delineating proteogenomic features of left‐ and right‐sided CRC within a Chinese cohort.

POLE, the catalytic subunit of DNA polymerase ε, governs DNA replication and repair fidelity [15]. Germline heterozygous variants in POLE's exonuclease or splice regions predispose to colorectal tumorigenesis [16]. Somatic POLE mutations induce hypermutated phenotypes independent of MMR status [17], associating with enriched CD8^+^ T‐cell infiltration, elevated cytotoxic T‐cell markers/effector cytokines, and improved immunotherapy response. Although occurring in only 1%–2% of CRC [18], POLE proofreading domain mutations reveal critical immunomodulatory functions. We further demonstrate that diminished POLE protein expression phenocopies the hypermutation effects of POLE mutations and inversely correlates with tumor mutational burden (TMB)—partially explaining sustained immunotherapy benefits in select pMMR/POLE wild‐type patients. Notably, POLE expression was significantly reduced in right‐sided relative to left‐sided CRC, a pattern recapitulated in normal adjacent tissues (NATs), reinforcing the embryologic and oncogenic divergence between tumor lateralities.

Conventional colorectal carcinogenesis follows two dominant pathways: the chromosomal instability (CIN) pathway, characterized by allelic losses at 5q (APC), 17p (TP53), and 18q (DCC/SMAD4) [19], and the MSI pathway. We identified 18q deletion as a mediator of metastatic progression exclusive to left‐sided CRC, aligning left‐sided tumors with CIN and right‐sided with MSI. The 18q locus harbors tumor suppressors (e.g., DCC, SMAD4) regulating apoptosis and proliferation; its deletion correlates with reduced overall survival and chemotherapy sensitivity in Stage II/III CRC [20, 21, 22]. Stage II patients without 18q deletion exhibit survival rates comparable to Stage I. However, conflicting reports question this association [23, 24], potentially attributable to unadjusted laterality effects. Current guidelines lack therapeutic criteria based on 18q status; prospective multicenter randomized trials are needed to evaluate its combined utility with tumor location for treatment escalation/de‐escalation in Stage II/III disease.

The predominance of dMMR/MSI‐H in right‐sided CRC is established [25]. Defective DNA damage repair amplifies replication errors [26], enhancing immunogenicity and immunotherapy efficacy [27]. Intriguingly, left‐sided CRC demonstrates stronger DNA repair activation than right‐sided across pMMR/MSS and dMMR/MSI‐H subgroups [28], yet right‐sided dMMR/MSI‐H metastatic CRC shows superior response to immune checkpoint blockade [8, 29]. While the right colon has been anecdotally proposed as immunologically privileged [30], underlying proteomic mechanisms remain undefined. Typically, dMMR causes uncorrected microsatellite errors culminating in MSI‐H. Strikingly, only 34.48% of left‐sided dMMR CRC progressed to MSI‐H versus 74.58% of right‐sided cases, with left‐sided tumors exhibiting significantly lower TMB. These findings suggest left‐sided dMMR CRC possess compensatory, MMR‐independent DNA repair systems functionally surpassing those in right‐sided tumors [28].

Unsupervised clustering stratified CRC into four subtypes (CC1–CC4) with distinct biology and therapeutic sensitivities. The CC1 subtype exhibited hyperproliferation and was centric to the MAPK pathway, suggesting potential vulnerability to MAPK inhibitors and anti‐EGFR monoclonal antibodies [31, 32]. CC2, enriched in right‐sided CRC, demonstrated a mucinous phenotype, which represented a hallmark of aggressive biology and chemoradiotherapy resistance [33, 34]. Targeting glycosyltransferases (critical for mucin synthesis) has shown promise in overcoming chemoresistance [35]. FOXA1 and HNF4G promote tumor cell growth and drug resistance in various solid cancers, including breast, prostate, pancreatic, and lung cancers [36, 37, 38, 39, 40]. We identified FOXA1 phosphorylation and HNF4G activation as upstream drivers of glycosyltransferase and mucins overexpression in CRC, nominating them as actionable targets for the mucinous subtype. CC3, also right‐sided‐enriched, harbored the highest immune infiltration and predicted responsiveness to immunotherapy. CC4, analogous to CC1 in left‐sided predominance, showed a high level of proliferative activity, uniquely exhibiting hyperactivation of RB1 kinase. Phosphorylated RB1 inversely correlated with apoptosis‐related gene sets, indicative of anti‐apoptotic reprogramming [9]. Mechanistically, CC4 tumors may evade apoptosis via RB1‐E2F axis‐mediated cell cycle dysregulation, suggesting heightened susceptibility to cytotoxic agents targeting cell cycle progression, such as 5‐fluorouracil and oxaliplatin. These insights recalibrate CRC classification beyond conventional TNM staging and CMS subtypes, advocating for a laterality‐integrated stratification framework to guide precision therapeutics. Further validation in prospective cohorts could redefine clinical algorithms, aligning CRC management with its multifocal biological complexity. Future efforts could develop simplified protein/phosphoprotein detection panels using immunohistochemistry or targeted mass spectrometry based on the distinct characteristics of each subtype. For example, detecting mucinous markers like MUC2 and MUC5AC to identify the mucinous CC2 subtype.

There were some limitations in the present study. First, a single‐center design potentially limiting representation of laterality‐associated heterogeneity. Second, our cohort exclusively utilized resected tumor samples from patients with nonmetastatic CRC, inherently limiting insights into metastatic progression. Furthermore, enrollment of treatment‐naïve patients. Future studies should incorporate targeted/immunotherapy‐exposed metastatic specimens, enabling mechanistic dissection of laterality‐linked differential responses to targeted therapy and immune checkpoint inhibitors. Lastly, while the use of bulk tumor tissues and matched NATs enhanced analytical depth and reproducibility in proteogenomic profiling, this approach may have compromised intratumoral heterogeneity assessment. Integrating multi‐region sampling strategies alongside spatially resolved single‐cell proteogenomics could refine spatial resolution and further validate our observations.

In summary, this study revealed differences in the origins and tumor microenvironments between left‐sided and right‐sided CRC through WES, proteomic, and phosphoproteomic sequencing, identified potential reasons for the differential efficacy of immunotherapy between the two, and proposed a novel proteomic molecular subtype that holds promise for precise diagnosis and treatment of CRC patients. The present study advances understanding of left‐ and right‐sided CRC that heretofore lacked comprehensive characterization based solely on single‐omics sequencing, and delineates actionable subtypes to inform precision interventions. These insights not only provide a roadmap for mechanistic and translational research but also advocate for the integration of CRC laterality into more precise and individualized treatment decision‐making.

## 4 Materials and Methods

### 4.1 Samples of Patients With CRC

#### 4.1.1 Sample Collection

The tumor and their matched NATs employed for the study were obtained from patients who underwent colorectal surgery at Peking University Shougang Hospital from January 2016 to June 2020, and who had not undergone any anticancer treatment prior to surgery. The surgical resection of the primary tumor and NATs (with a distance of more than 5 cm from the tumor margin) were promptly placed in cryopreservation tubes and transferred to liquid nitrogen within 20 min. A total of 80 paired specimens were collected, along with the relevant clinical information (Table S1). Patients diagnosed with hereditary CRC, such as Lynch syndrome and familial adenomatous polyposis, as well as Stage IV and those with a previous history of malignancy, were excluded from participation. Right‐sided CRC was located proximally at the splenic flexure of the transverse colon, and left‐sided CRC was located distally at the splenic flexure and beyond the extent of the transverse colon. The collection of all patient samples was conducted in accordance with the directives set forth by the hospital research ethics committee, and written informed consent was obtained from each participant.

### 4.2 WES, Proteomic, and Phosphoproteomic Analysis

Sequencing and analysis of WES, proteome, and phosphoproteome samples are described in the Supporting Information Methods.

### 4.3 Effects of CNAs

Spearman's correlations between CNA values (gene level) and protein/phosphoproteomic abundances were calculated. CNAs with significant correlation with proteins were selected based on an FDR of less than 0.05. Correlations were visualized using the R package multiOmicsViz (v1.20.0). The impact of 18q deletion was analyzed by a gene set enrichment analysis (GSEA) method.

### 4.4 GSEA

GseaVis was used to perform GSEA [41], based on KEGG, Wikipathways, and Hallmarks gene sets downloaded from MSigDB v7.1 (http://software. broadinstitute.org/gsea/msigdb/index.jsp) [42]. A *p*‐value less than 0.05 was used as a cutoff. The normalized enrichment score was used to reflect the degree of pathway overrepresentation.

### 4.5 Survival Analysis

Overall survival (OS) was defined from the date of CRC surgery to the date of death or last available follow‐up. Prior to the log‐rank test of a given protein, the survminer 0.4.9 R package with maxstat (maximally selected rank statistics) was used to determine the optimal cutpoint for the selected samples according to the previous study [43]. OS curves were then calculated (Kaplan–Meier analysis, log‐rank test) based on the optimal cutpoint.

### 4.6 CT Antigen Analysis

CT antigens were downloaded from the CTdatabase [44], which consists of 269 CT antigens with carefully curated and annotated literature‐derived information. Identified CT antigens overexpressed by at least two‐fold in tumors compared to NATs in more than 5% of all samples.

### 4.7 Estimation of CD274 Levels

We calculated all Pearson correlations of genes, both identified in this study and in the CPTAC CRC cohort, with CD274 levels. A linear model was conducted using genes with a Pearson's correlation greater than 0.65. Pearson's correlation between true CD274 and estimated CD274 levels was 0.86 in the CPTAC test set and 0.83 in the TCGA cohort. CPTAC CRC cohort data were downloaded from LinkedOmics [45]. The TCGA CRC cohort data were downloaded from Xena [46] before January 2025.

### 4.8 Molecular Subtyping of CRC Tumors

Consensus clustering was conducted using the R package ConsensusClusterPlus (v.1.60.0) [47] using Pearson's correlation as the distance measure. The number of repetitions was set as 1000 bootstraps; the item subsampling proportion was set as 0.8; the feature subsampling proportion was set as 1. Proteins with the top 25% highest variance in tumor samples were used for partitioning around medoids clustering with up to six groups. Consensus matrices for *k* = 2, 3, 4, 5, 6 clusters are shown in Figure S5A. The consensus matrix for *k* = 4 showed clear separation among clusters. The cumulative distribution function of the consensus matrix for each *k*‐value was also measured. The relative change in area under the cumulative distribution function curve and the average silhouette distance was applied for *k*‐value selection (Figure S5B,C).

### 4.9 Causal Interaction Analysis

CausalPath [48] analysis was performed to visualize the causal interactions of the differentially expressed proteins (DEPs) and phosphosites in the CC1 subtype.

### 4.10 Tumor Microenvironment Analysis

Immune score, stromal score, and tumor purity were inferred using the R package IOBR [49] and the ESTIMATE algorithm [50]. To evaluate the tumor immune microenvironment of CRC tumors, the raw enrichment scores of 64 different cell types were computed via xCell [51], based on the tumor proteomic profiles. Cell types (such as hepatocytes, neurons, and astrocytes, etc.) that did not exist in CRC or tumor adjacent tissues were excluded.

### 4.11 Multiplex Immunofluorescence Staining

Consecutive 4‐µm FFPE sections were dewaxed (xylene), rehydrated (ethanol gradient), and subjected to epitope retrieval using EDTA‐based buffer (pH 9.0, 95°C, 20 min). Endogenous peroxidase activity was quenched with 3% H_2_O_2_ (10 min), followed by protein block (BSA 10%, RT, 30 min). Primary antibodies for PanCK, CD34, CD68, and CD168 were used. The primary antibody incubation, secondary antibody incubation, and PANO TSA staining were repeated until all the markers were stained. Finally, the slides were stained with DAPI. The stained slides were scanned using the High‐content Screening Analysis System (ImageXpressMicro Confocal, Molecular Devices), and positive cells were quantified using ImageJ software.

### 4.12 Single‐Cell RNA Sequencing (scRNA‐seq) Data Processing and Analysis

The scRNA‐seq data used in this study were downloaded from the GEO database under the accession code GSE236581. The R package Seurat (version 4.2.1) was used to analyze the scRNA‐seq data. Cells [1] with less than 600 or more than 25,000 UMI counts [2], less than 600 detected genes, or [3] more than 5% (70% for CD45^−^ cells) mitochondrial gene counts, were filtered out. The Seurat‐implemented NormalizeData function was used to perform the library‐size correction and logarithm transformation, with the obtained expression matrix used for downstream analyses. Major cell types and subtypes were annotated by comparing the typical marker genes and differentially expressed genes in each cluster.

### 4.13 Statistical Analysis

Statistical analysis was performed under the R programming environment (https://www.r‐project.org/) in version 3.5.2. Comparison between groups was examined either by Student's *t*‐test, nonparametric test, or ANOVAR. Benjamini–Hochberg procedure was used to adjusted *p*‐values for multiple hypothesis testing when appropriate.

## Author Contributions

Conceptualization: J.G., X.W., and M.L. Data curation: A.H., Y.S., and Z.C. Methodology: Z.C., and H.C. Formal analysis: A.H., J.X., and H.H. Resources: Z.G. Writing – original draft preparation: A.H., Y.S., J.C., and Y.Y. Writing – review and editing: H.C., X.W., M.L., and J.G. Funding acquisition: J.G. All authors commented on the study and approved the final manuscript.

## Funding

This study was funded by the National Natural Science Foundation of China (Grant Number: 82073223), the Capital's Funds for Health Improvement and Research of China (Grant Number: CFH 2024‐1‐6041), and the Young Investigator Training Program of the First Affiliated Hospital of Nanchang University (Grant Number: YFYPY202572).

## Ethics Statement

This study was conducted in accordance with all ethical guidelines concerning human experimentation described in the Declaration of Helsinki and was approved by the Institutional Review Board of Peking University Shougang Hospital (IRB‐SOP‐21‐00). All patients provided written informed consent.

## Conflicts of Interest

Zhongyi Chen is an employee of Jingjie PTM BioLab Co. Ltd., but has no potential relevant financial or non‐financial interests to disclose. The remaining authors declare no conflicts of interest.

## Supporting information




**Supporting Information**: mco270806‐sup‐0001‐SuppMat.docx

## Data Availability

The datasets used and/or analyzed during the current study are available from the corresponding author upon reasonable request.
