# Supplementary material for: Integrative Proteogenomic Characterization of Left‐Sided and Right‐Sided Colorectal Cancer
Source: MedComm (2020). 2026 Jun 8;7(6):e70806. doi: 10.1002/mco2.70806 (PMC13243882; doi:10.1002/mco2.70806)
Supplement: Supplementary file 1 — Supporting Information: mco270806‐sup‐0001‐SuppMat.docx [file MCO2-7-e70806-s001.docx]

**Supplementary methods**

**Whole-exome sequencing (WES)**

***DNA extraction and sequencing***

Total DNA from tumor and NATs was extracted using the QIAamp DNA mini kit (QIAGEN, Hilden, Germany) according to the manufacturer's instructions, and agarose gel electrophoresis was used to analyze the extent of DNA degradation and the presence of RNA contamination. DNA samples were assayed for purity and concentration using NanoDrop. DNA was sheared using dsDNA fragmentase (New England BioLabs, Inc., Ipswich, MA, USA). dsDNA fragments (150-250 bp) were then size-selected using Ampure XP beads (Beckman Coulter, Inc., Brea, CA, USA) for DNA fragments (150-250 bp). DNA fragments were used for library construction using the KAPA Library Preparation Kit (Kapa Biosystems, Inc., Wilmington, MA, USA) according to the manufacturer's protocol. All purification steps were performed using Agencourt AMPure XP beads (Beckman Coulter, Inc., Brea, CA, USA). End repair and 3’ terminal A-tailing were carried out after the DNA was fragmented. After passing the library check, the exonic regions of the DNA were enriched with the updated SuperWES gene panel for standard whole-genome sequencing (HaploX Biotechnology, Shenzhen, China), and the different libraries were pooled according to the effective concentration and the desired amount of downstream data required and subjected to Illumina PE150 sequencing.

***Data processing***

Preprocessing the Raw Data obtained from the Illumina sequencing platform using fastp (V0.12.6, <https://github.com/OpenGene/fastp>) the Clean Data for subsequent analysis. The specific processing steps are as follows:1) adapter trimming; 2) remove the reads in which the N base has reached a certain percentage (default length of 5 bp); 3) remove the reads which contain low quality bases (default quality threshold value ≤ 20) above a certain portion (default 40%); 4) sliding window trimming: the bases in the sliding window (default is 4bp) with mean quality below cutting quality (default is 20) will be cut;

Sequencing reads were aligned to the reference genome (hg19plus) using Sentieon BWA (https://www.sentieon.com) with default parameters. Subsequent processing, including sort, duplicate removal was proformed using Sentieon driver (<https://www.sentieon.com>). Finally, based on mapping results obtained depth, coverage using bamdst (https://github.com/shiquan/bamdst).

***Variant detection and annotation***

The raw SNP/Indel sets are called by Sentieon DNAseq (https://support.sentieon.com/manual/DNAseq_usage/dnaseq/). The Somatic SNP/Indel are called by Mutect2 (https://software.broadinstitute.org/gatk/documentation/article?id=11077). The Somatic CNV are called by Control-FREEC (http://boevalab.com/FREEC/). ANNOVAR (http://annovar.openbioinformatics.org/en/latest/) was used for functional annotation of variants.

***Maftools***

Maftools (v 2.12.0)(1) was used for visulazition of the top 20 most frequently mutated genes in this cohort.

***Mutational signatures***

The non-negative matrix factorization algorithm of Sigminer(2) was used to exact mutational signatures of tumor samples. Cosine similarities were calculated between the decomposed signatures and signatures derived from COSMIC (https://cancer.sanger.ac.uk/cosmic)(3).

**Proteomic and phosphoproteomic analysis**

***Protein extraction***

Remove the samples from the liquid nitrogen, weigh the sample into a mortar pre-cooled with liquid nitrogen, and grind the sample well with liquid nitrogen until it is powdered. Transfer the powder to a 5 ml centrifuge tube. Add 4 times the volume of pre-cooled 10% TCA/acetone (TCA: Sigma-Aldrich, T4885; acetone: Hangzhou Hannao Chemical) to the powder and precipitate at -20℃ for 4 hours. After centrifugation at 4,500g for 5 minutes at 4℃, the supernatants were discarded, and the pellet was washed three times with chilled acetone, air dried, and fourfold volume of lysis buffer (1% SDS, 1% protease inhibitor, 1% phosphatase inhibitor) (Merck Millipore, 539137) was added to the protein precipitate, followed by sonication on ice for 3 minutes using a high-intensity ultrasonic processor ( Scientz) (Ningbo Xinzhi Bioscience Co. , Ltd., XM-900T) for three minutes on ice. The remaining residue was removed by centrifugation at 4℃, 12000g for 10 minutes. The supernatant was collected and the protein concentration was determined using a BCA kit (Beyotime, P0011) according to the manufacturer's instructions.

***Tryptic digestion***

An equal amount of each sample protein was taken for tryptic digestion, the volume was adjusted to consistency with the lysate, 1x volume of pre-cooled acetone was added, vortexed and mixed, then 4x volume of pre-cooled acetone was added and precipitation was performed at -20°C for 2h. Centrifugation was performed at 4500g for 5 minutes, the supernatant was discarded and the precipitate was washed 2-3 times with pre-cooled acetone. After drying of the precipitate, tetraethyl ammonium bromide (TEAB, Sigma-Aldrich, T7408) was added to a final concentration of 200 mM, the precipitate was solubilized by sonication, and trypsin (Promega, V5117) was added at a ratio of 1:50 (protease:protein, m/m) and digested overnight. Dithiothreitol (DTT) (Sigma-Aldrich, D9163) was added to a final concentration of 5 mM, and the solution was reduced at 37°C for 60 minutes. Iodoacetamide (IAA) (Sigma-Aldrich, V900335) was then added to a final concentration of 11 mM, and the solution was incubated for 45 minutes at room temperature in the dark.

***The enrichment of phosphorylated peptide***

Peptides were dissolved in enrichment buffer solution (50% acetonitrile/0.5% acetic acid), and the supernatant was transferred to prewashed immobilized metal ion affinity chromatography (IMAC) material (ThermoFisher Scientific, A32992) and placed on a rotary shaker for incubation with gentle shaking. At the end of the incubation, the material was washed three times in succession with 50% acetonitrile/0.5% acetic acid and 30% acetonitrile/0.1% trifluoroacetic acid buffer solutions. Finally, the phosphopeptides were eluted with 10% ammonia, and the eluate was collected and lyophilized under vacuum. After extraction, the material was desalted according to the C18 ZipTips instructions and lyophilized under vacuum for liquid mass spectrometry (MS) analysis.

***MS***

The liquid chromatography mobile phase consisted of solvent A (0.1% formic acid, 2% acetonitrile/water) and solvent B (0.1% formic acid, 90% acetonitrile/water). The peptides were dissolved in solvent A and loaded directly onto a home-made reversed-phase analytical column (25 cm long, 100 µm internal diameter). The peptides were separated using the following gradients: 0-68 minutes, 6%-23% B; 68-82 minutes, 23%-32% B; 82-86 minutes, 32%-80% B; 86-90 minutes, 80% B. All gradients were run on an EASY-nLC 1200 UPLC system (ThermoFisher Scientific, Waltham, USA) at a constant flow rate of 500 nl/minutes. The isolated peptides were analyzed in an Orbitrap Exploris 480 nanoelectrospray ion source. The electrospray voltage was 2300 V. The FAIMS compensation voltages (CV) were set at -45 V, -65 V. Both the peptide parent ions and their secondary fragments were analyzed using a high-resolution Orbitrap detector (ThermoFisher Scientific, Waltham, USA). The primary MS scan range was set to 400-1200 m/z and the scan resolution was set to 60,000; the secondary MS scan range was set to a fixed start point of 110 m/z, the secondary scan resolution was set to 15,000, and TurboTMT was set to off. The data acquisition mode used a data dependent acquisition (DDA) procedure, i.e. after the primary scan, the pre-peptide parent ions with the highest signal intensities were selected to sequentially enter the HCD collision cell to be fragmented at 27% fragmentation energy, and the same sequential secondary mass spectrometry analysis was performed. To improve the effective utilization of the mass spectrometry, the automatic gain control (AGC) was set to 100%, the signal threshold was set to 50000 ions/s, the maximum injection time was set to Auto, and the dynamic exclusion time for tandem mass spectrometry scans was set to 20 s to avoid repeated scans of the parent ions.

For phosphorylated proteomic samples, the steps were similar to those for proteomic samples except that the peptide separation gradient was as follows: 0-70 min, 3%-20% B; 70-82 min, 20%-30% B; 82-86 min, 30%-80% B; and 86-90 min, 80% B. In addition, the secondary scan resolution was set to 30,000, the AGC was set to 75%, the signal threshold was set to 20000 ions/s, the maximum injection time was set to 100 ms, and the dynamic exclusion time for tandem mass spectrometry scans was set to 30 s to avoid repetitive scans of parent ions.

***MS platform quality control***

For quality control (QC) of MS performance, tryptic digests of HeLa cell lysates were measured as a QC standard every 1 or 2 days. The HeLa cell line, obtained from ATCC, was authenticated by short-tandem repeat profiling and was tested negative for mycoplasma contamination. Pairwise Spearman’s correlation coefficients were calculated and shown in **Figure S1A-S1B**.

***Preprocessing of proteomic, phosphoproteomic data***

Proteome quantification was conducted using the iBAQ algorithm, and phosphoproteome was quantified at site level. All quantification data were conducted quantile normalization and log2 transformation. Proteins, phosphosites having more than 50% missing data in both tumor and NAT samples were excluded for further analysis. K-nearest neighbor (KNN) imputation was applied to impute the missing values using R package DreamAI.

**Differential expressed protein**

Differential expressed protein was analyzed based on Wilcoxon rank-sum test. Benjamini–Hochberg method was used for p value adjustment. Fold change > 1.5 was set as the threshold. The DEPs was used for overrepresentation analysis using DAVID(4) and ConsensusPathDB(5).

**Kinase activity analysis**

The kinase activity inference was based on the kinase-substrate relationships obtained from PhosphoSitePlus and NetworKIN(6). The predicated substrates with a NetworKIN score＞5 were remained. Kinase activities in each samples was calculated using the ssGSEA method(7) in the GSVA package(8). KSEA was also performed using the PhosphoSitePlus and NetworKIN database using the KSEA app (https://casecpb.shinyapps.io/ksea/)(9). Kinase with FDR < 0.05 were considered significant.

**Transcription factor activity analysis**

The transcription factor activity inference was based on the TF-target gene relationships obtained from DoRothEA(10). The TF-target gene relationships with A, B, C confidences were remained. TF activities in each sample was calculated using the VIPER(11). ChEA3(12) was used to analysis the unstream TF of genes involved in Mucin type O-glycan biosynthesis.

**Cancer-testis (CT) antigen analysis**

CT antigens were downloaded from the CTdatabase(13), which consists of 269 CT antigens with carefully curated and annotated literature-derived information. Identified CT antigens over-expressed by at least 2-fold in tumors compared to NATs in more than 5% of all samples.

**Causal interaction analysis**

CausalPath(14) analysis was performed to visualization the causal interactions of the differentially expressed proteins and phosphosites in CC1 subtype.

**Tumor microenvironment analysis**

Immune score, stromal score and tumor purity were inferred using the R package, ESTIMATE (v1.0.11)(15). To evaluate the tumor immune microenvironment of CRC tumors, the raw enrichment scores of 64 different cell types were computed via xCell(16), based on the tumor proteomic profiles. Cell types (such as hepatocytes, neurons, and astrocytes et al.) did not exist in CRC or tumor adjacent tissues were excluded.

**Tissue isolation and organoid/tumoroid culture**

Tissue isolation and organoid/tumoroid culture were performed in accordance with our previous study(17). The RNA-seq data have been deposited in the GEO database(GSE261004).

**Statistical analysis**

Statistical analysis was performed under R programming environment (https://www.r-project.org/) in version 3.5.2. Comparison between groups were examined either by Student’s t-test, nonparametric test or ANOVAR. Benjamini-Hochberg procedure was used to adjusted P values for multiple hypotheses testing when appropriate.

**References**

1. Mayakonda A, Lin DC, Assenov Y, Plass C, Koeffler HP. Maftools: efficient and comprehensive analysis of somatic variants in cancer. Genome Res. 2018;28(11):1747-1756.

2. Wang S, Tao Z, Wu T, Liu XS. Sigflow: an automated and comprehensive pipeline for cancer genome mutational signature analysis. Bioinformatics. 2021;37(11):1590-1592.

3. Tate JG, Bamford S, Jubb HC, et al. COSMIC: the Catalogue Of Somatic Mutations In Cancer. Nucleic Acids Res. 2019;47(D1):D941-d947.

4. Huang da W, Sherman BT, Lempicki RA. Systematic and integrative analysis of large gene lists using DAVID bioinformatics resources. Nat Protoc. 2009;4(1):44-57.

5. Kamburov A, Pentchev K, Galicka H, Wierling C, Lehrach H, Herwig R. ConsensusPathDB: toward a more complete picture of cell biology. Nucleic Acids Res. 2011;39(Database issue):D712-717.

6. Linding R, Jensen LJ, Pasculescu A, et al. NetworKIN: a resource for exploring cellular phosphorylation networks. Nucleic Acids Res. 2008;36(Database issue):D695-699.

7. Barbie DA, Tamayo P, Boehm JS, et al. Systematic RNA interference reveals that oncogenic KRAS-driven cancers require TBK1. Nature. 2009;462(7269):108-112.

8. Hänzelmann S, Castelo R, Guinney JJBB. GSVA: Gene set variation analysis for microarray and RNA-Seq data. 2013;14(1):7.

9. Wiredja DD, Koyutürk M, Chance MR. The KSEA App: a web-based tool for kinase activity inference from quantitative phosphoproteomics. Bioinformatics. 2017;33(21):3489-3491.

10. Garcia-Alonso L, Holland CH, Ibrahim MM, Turei D, Saez-Rodriguez J. Benchmark and integration of resources for the estimation of human transcription factor activities. Genome Res. 2019;29(8):1363-1375.

11. Alvarez MJ, Shen Y, Giorgi FM, et al. Functional characterization of somatic mutations in cancer using network-based inference of protein activity. Nat Genet. 2016;48(8):838-847.

12. Keenan AB, Torre D, Lachmann A, et al. ChEA3: transcription factor enrichment analysis by orthogonal omics integration. Nucleic Acids Res. 2019;47(W1):W212-w224.

13. Almeida LG, Sakabe NJ, deOliveira AR, et al. CTdatabase: a knowledge-base of high-throughput and curated data on cancer-testis antigens. Nucleic Acids Res. 2009;37(Database issue):D816-819.

14. Babur Ö, Luna A, Korkut A, et al. Causal interactions from proteomic profiles: Molecular data meet pathway knowledge. Patterns (N Y). 2021;2(6):100257.

15. Yoshihara K, Shahmoradgoli M, Martínez E, et al. Inferring tumour purity and stromal and immune cell admixture from expression data. Nat Commun. 2013;4:2612.

16. Aran D, Hu Z, Butte AJ. xCell: digitally portraying the tissue cellular heterogeneity landscape. Genome Biol. 2017;18(1):220.

17. Xiong L, Xu Y, Gao Z, et al. A patient-derived organoid model captures fetal-like plasticity in colorectal cancer. Cell Res. 2025;35(9):642-655.


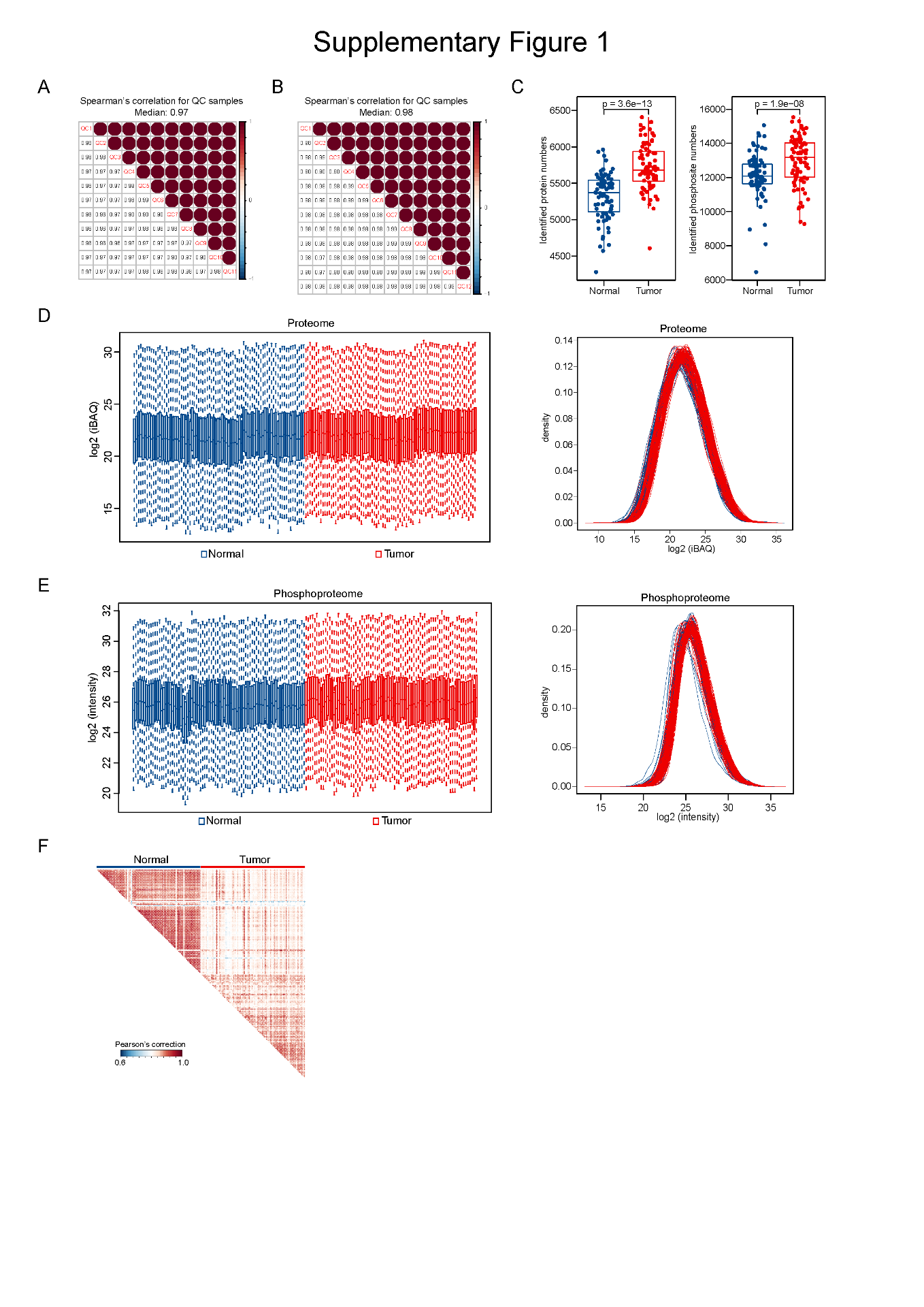


**Figure S1. Overview of proteomic and phosphoproteomic profiles.**

1. Heatmap of correlation coefficients for proteomics QC samples. Correlation analysis was performed on 11 HeLa cell samples as a proteomics QC to assess the stability of label-free quantification. Lower left half panel: pairwise calculation of Spearman correlation coefficients between the 11 samples. Upper right half panel: pairwise comparison of the 11 samples by means of a scatter plot. An average correlation coefficient of 0.97 was observed.
2. Heatmap of correlation coefficients for phosphoproteomics QC samples. Correlation analysis of 12 HeLa cell samples was performed as a phosphoproteomics QC to assess the stability of label-free quantification. Lower left half panel: pairwise calculation of Spearman correlation coefficients between the 12 samples. Upper right half panel: pairwise comparison of the 12 samples by means of a scatter plot. An average correlation coefficient of 0.98 was observed.
3. Box plots of proteins identified in tumors (red) and adjacent tissues (blue) (n = 80 pairs). P value is derived from two-sided paired t test.
4. Boxplots and density curves of the log2 (iBAQ) protein expression levels for the 80 pairs of tumors (red) and NATs (blue).
5. Boxplots and density curves of the log2 (intensity) phosphosites expression levels for the 80 pairs of tumors (red) and NATs (blue).
6. Pearson’s correlation of tumor and adjacent tissues based on proteome data. The mean correlation was 0.863 for the 80 tumor samples and 0.905 for the 80 NATs.


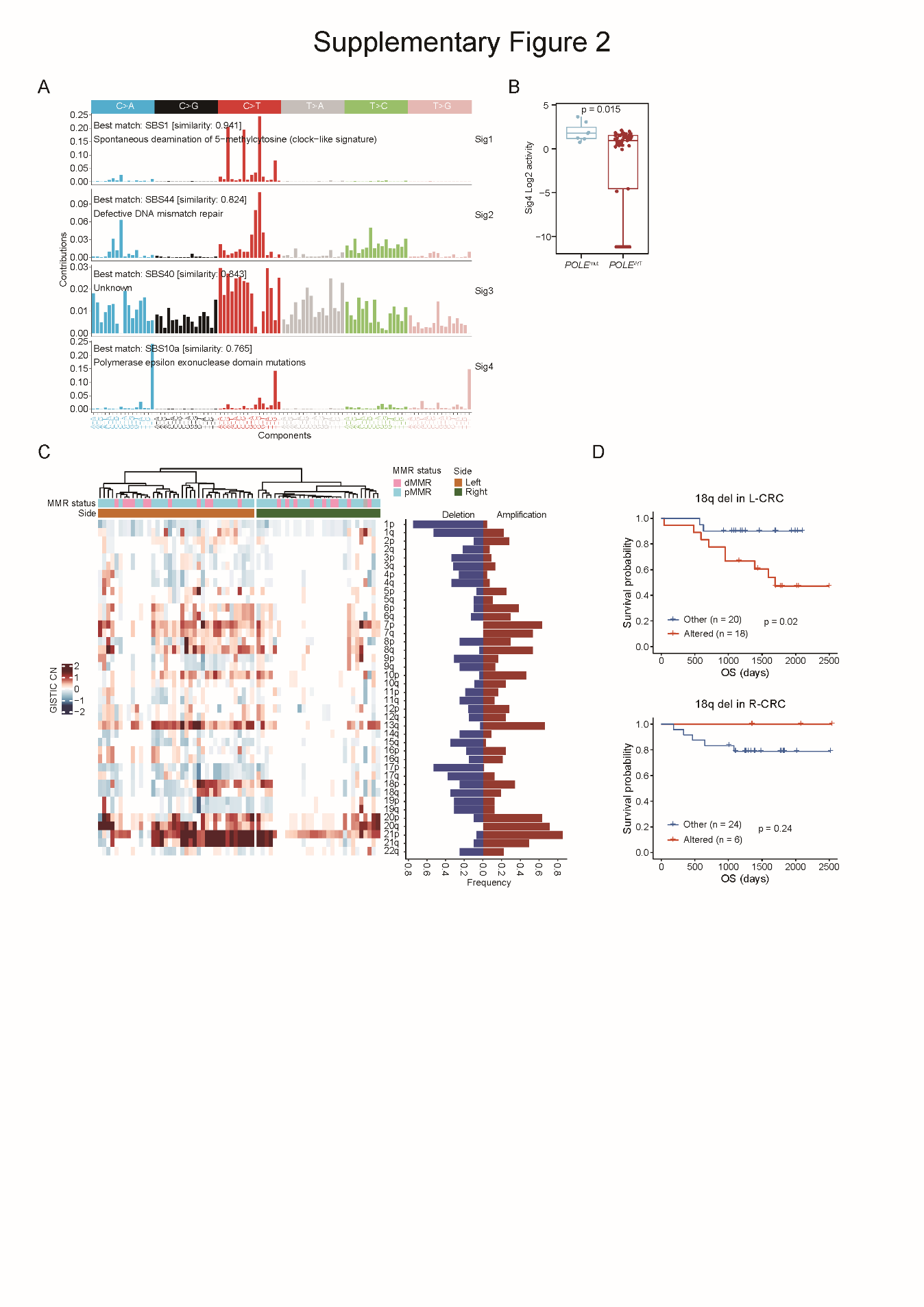


**Figure S2. Somatic alterations and mutational profiles of Chinese left-sided and right-sided CRC.**

1. Decomposition of four mutational signatures from the side-based CRC cohort.
2. Comparion of Sig4 activity between *POLE*^mut^ (blue, n=7) and *POLE*^wt^ (red, n=61) CRC samples. Sig4 activity of patients with *POLE*^wt^ was significantly stronger than that of *POLE*^wt^ (P values were derived from Wilcoxon rank-sum test)。
3. Arm-level CNA copy numbers in CRC samples (Left panel); Deletion and Amplication frequencies (Right panel). A total of 25 chromosome-level SCNA events, 6336 gene-level SCNA gain events (26.10%), and 7600 gene-level losses (31.31%) were identified.
4. Kaplan–Meier curves of OS for patients with or without 18q deletion in Left-CRC (above) and Right-CRC (below) (P values were derived from log-rank test).


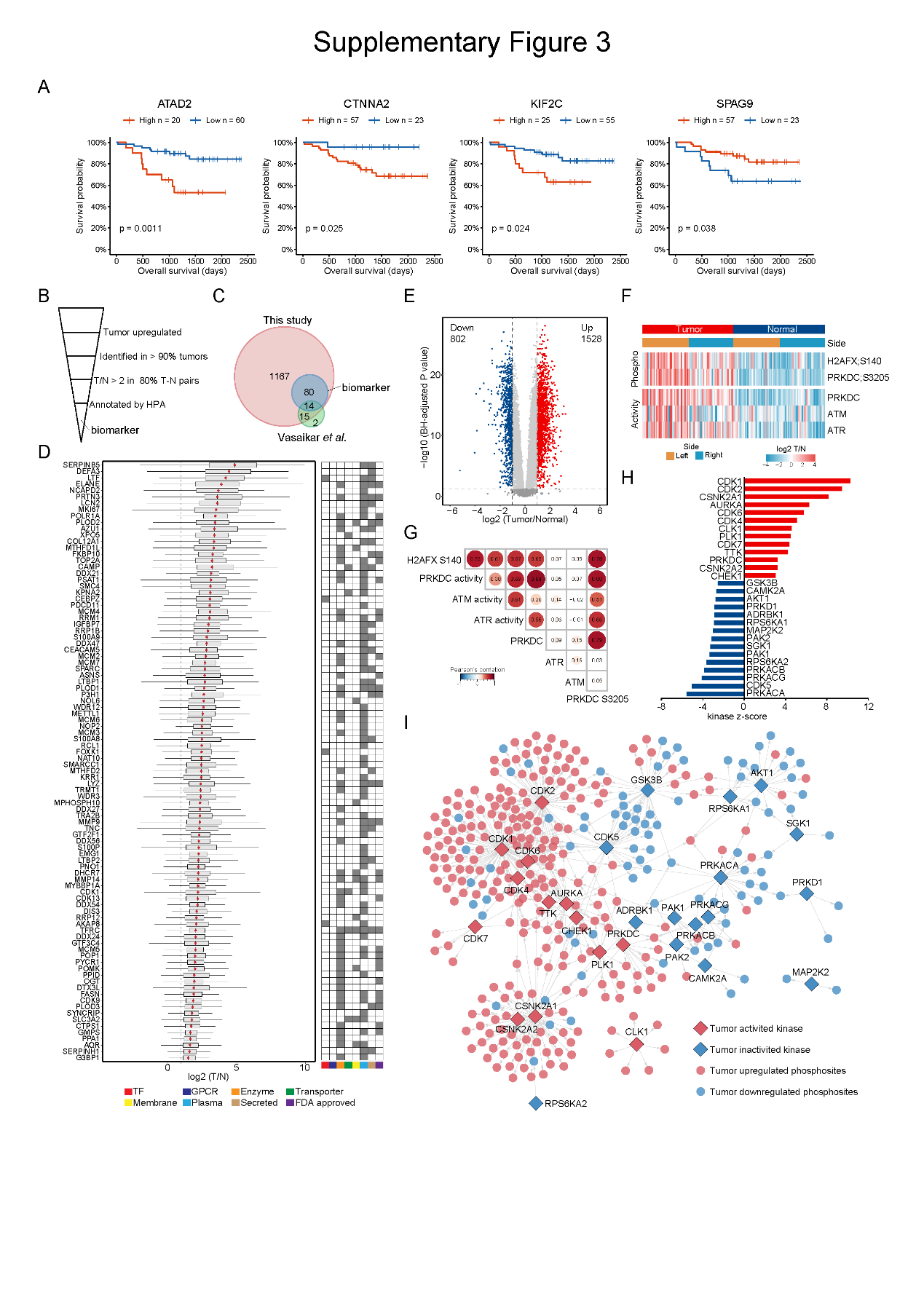


**Figure S3. Proteomics and phosphoproteomics landscape of CRC.**

1. Association between CT-antigens protein expressions and OS. Among the 9 identified CT antigens, ATAD2, CTNNA2, KIF2C, and SPAG9 were significantly associated with OS in CRC patients (two-sided log-rank test).
2. Process for screening potential biomarkers in CRC. The cancer biomarkers were screened out by following rules: tumor-upregulated proteins; identified in 90% of tumor samples; with >2-fold increase in more than 80% of T/N pairs; potential clinical utilities defined by HPA database.
3. Potential biomarkers of CRC. Venn plot showing the upregulated proteins in this study (red), potential cancer biomarkers of CRC identified in this study (blue) and Vasaikar’s study (green).
4. Log2 FC between tumor and matched NATs (n = 80) is shown for the CRC biomarkers. Boxplots show the median (central line), the 25–75% IQR (box limits), the ±1.5 × IQR (whiskers). The foldchanges of these biomarkers in tumor versus NATs (left panel). The functions of these biomarkers (right panel). These biomarkers are annotated with potential clinical utilities by HPA.
5. Volcano plot showing differentially expressed phosphosites (two-sided paired t test, Benjamini–Hochberg-adjusted p value < 0.05, foldchange > 2) in tumor and adjacent tissues. Proteins that were significantly overexpressed in tumor/adjacent tissues are presented with red/blue filled scatters.
6. Heatmap showing the widely DNA damage in CRC samples. H2FAX S140 and PRKDC S3205 phosphorylation and kinase PRKDC, ATM, and ATR activities were significantly increased in tumor tissues, especially left-sided CRC.
7. Correlations of H2AFX S140 (γ-H_2_AX, DNA double-strand break marker) and DNA double-strand break repair related kinases. PRKDC activity correlated most strongly with H2AFX S140.
8. Differential analysis of kinase activities by KSEA between tumor and adjacent tissues. Red, kinases with increased activity in tumor tissues; blue, kinases with decreased activity in tumor tissues.
9. Kinase-substrate interaction in CRC tumors. Red diamonds represent kinases that are activated in the tumor and red circles represent phosphorylation sites that are upregulated in the tumor. Blue diamonds represent kinases that are inactivated in the tumor and red circles represent phosphorylation sites that are downregulated in the tumor.


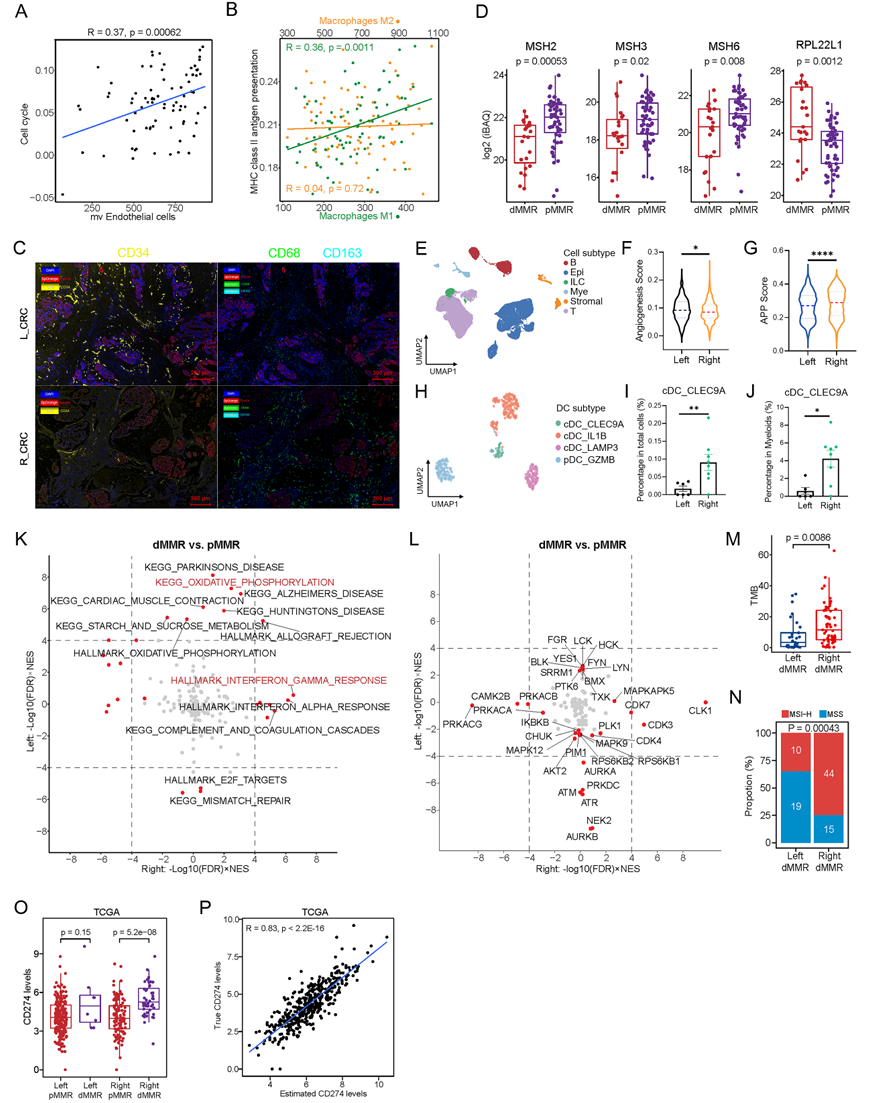


**Figure S4. Differences in proteomics and phosphoproteomics between right-sided and left-sided CRC.**

1. Correlation analysis between cell cycle GSVA scores and mv Endothelial cells (Spearman’s correlation).
2. Correlation analysis between MHC class II antigen presentation GSVA scores and macrophages. Green, M1 macrophages. Orange, M2 macrophages (Spearman’s correlation).
3. Boxplots showing the differences of MSH2, MSH3, MSH6, and RPL22L1 between dMMR-CRC (red, n=23) and pMMR-CRC (purple, n=56) (Wilcoxon rank-sum test).
4. Multiplex immunofluorescence staining for CD34^+^, CD68^+^, and CD163^+^ cells in left-sided and right-sided CRC. Green: DAPI. Red: PanCK. Yellow: CD34. Green: CD68. Aqua: CD163.
5. UMAP plot of 63,433 cells from 14 patients of dMMR CRC in the GSE236581 cohort.
6. Comparison of angiogenesis score between left-sided (n=6) and right-sided (n=8) dMMR CRC. *, p<0.05.
7. Comparison of antigen processing and presentation (APP) score between left-sided (n=6) and right-sided (n=8) dMMR CRC. ****, p<0.0001.
8. UMAP plot of dendritic cells in the GSE236581 cohort.
9. Comparison of CLEC9A^+^ dendritic cells between left-sided (n=6) and right-sided (n=8) dMMR CRC in total 63,433 cells. **, p<0.01.
10. Comparison of CLEC9A^+^ dendritic cells between left-sided (n=6) and right-sided (n=8) dMMR CRC in myeloid cells. *, p<0.05.
11. Comparison of Hallmarks and KEGG pathways between dMMR and pMMR in left-sided and right-sided CRC tumors using GSEA.
12. Comparison of kinase activities between dMMR and pMMR in left-sided and right-sided CRC tumors using GSEA.
13. Comparison of TMB between left-sided dMMR CRC (blue, n=29) and right-sided dMMR CRC (red, n=59) tumors in the SYSUCC CRC cohort (PMID: 35487942) (two-sided unpaired t test).
14. Bar plots showing the distribution of MSI-H (red) and MSS (blue) among between left-sided dMMR and right-sided dMMR CRC tumors (PMID: 35487942) (Fisher’s exact test).
15. Comparison of CD274 levels between dMMR (blue) and pMMR (red) tumors in both left-sided and right-sided CRC (two-sided unpaired t test) in the TCGA cohort.
16. Correlation analysis between CD274 levels and CD274 level estimated by linear regression (Spearman’s correlation).


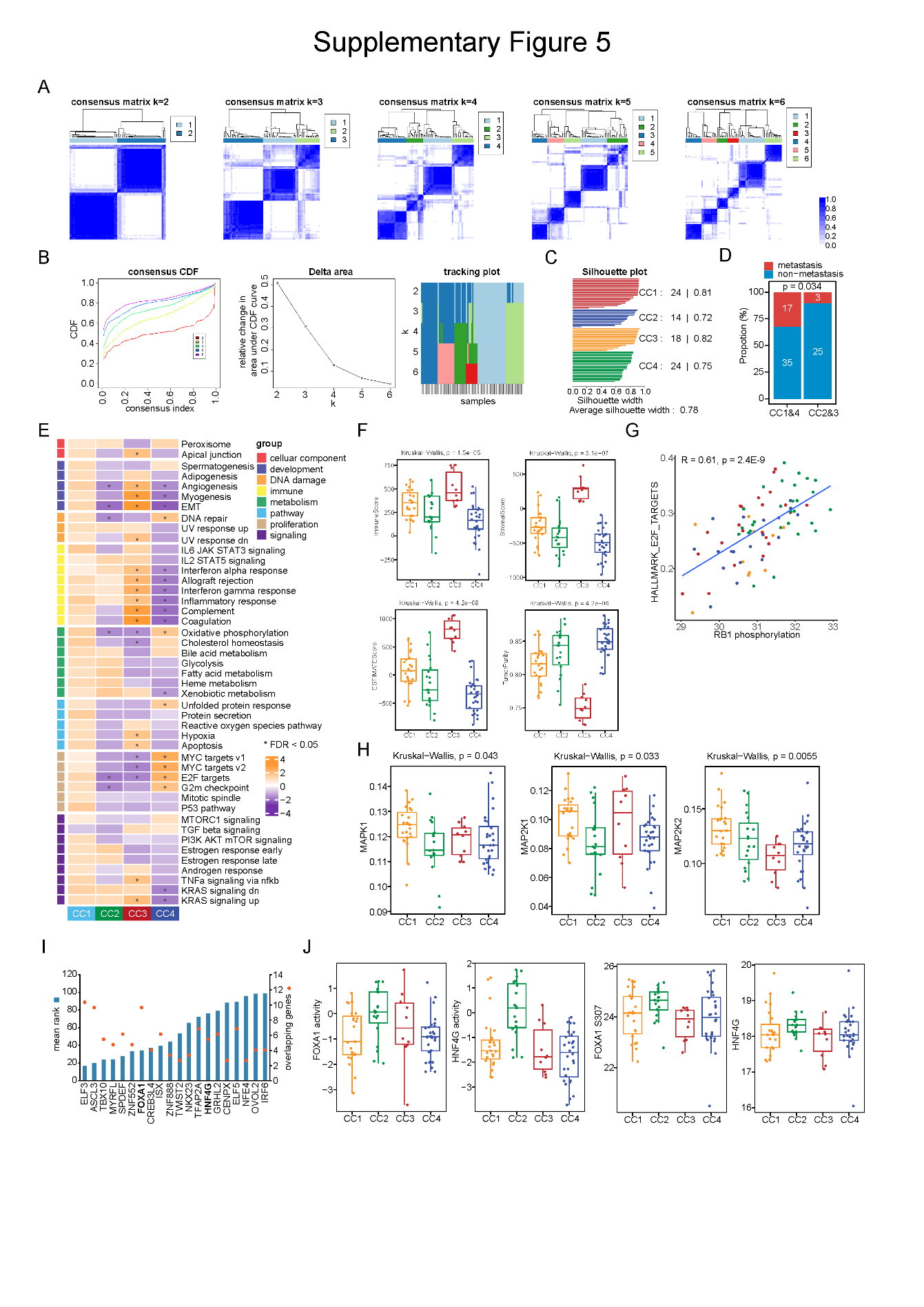


**Figure S5.** **Proteomics based construction of molecular subtypes and their characteristics.**

1. Consensus matrices of the 80 CRC tumor samples from k = 2 to k = 6.
2. Cumulative distribution function plot, delta plot, and tracking plot corresponding to consensus matrices from k = 2 to k = 6.
3. Silhouette plot (k = 4).
4. Bar plots showing the distribution of metastasis and non-metastasis tumors between CC1&4 and CC2&3 (Fisher’s exact test).
5. Heatmap showing GSEA NES of Hallmarks among proteomic subtypes. Hallmarks pathways with p < 0.05 were labeled by asterisk.
6. Boxplots showing the differences of immune score, stromal score, ESTIMATE score, and tumor purity among subtypes (Kruskal-Wailis test). CC1: n=23. CC2: n=19. CC3: n=10. CC4: n=28.
7. Correlation analysis between RB1 phosphorylation and Hallmark E2F targets (Spearman’s correlation).
8. Boxplots showing the differential kinase activities of MAPK1, MAP2K1, and MAP2K2 among subtypes (Kruskal-Wailis test). CC1: n=23. CC2: n=19. CC3: n=10. CC4: n=28.
9. CHEA3 analysis of unstream TF (top 20) of genes involved in Mucin type O-glycan biosynthesis.
10. Boxplots showing the differences of FOXA1 TF activities, FOXA1 S307 phosphorylation abundances, HNF4G TF activities, and HNF4G protein abundances among subtypes (Kruskal-Wailis test). CC1: n=23. CC2: n=19. CC3: n=10. CC4: n=28.

**Supplementary table 1. Clinical characteristics of the included patients.**

| Sample | Sex | Age | Tumor location | AJCC stage (8th) | Primary tumor | MMR status | Postoperative chemotherapy | Postoperative chemotherapy regimen | Recurrence /metastasis | Time to recurrence /metastasis or last follow-up (months) | Survival status | Time to dead or last follow-up (months) |
| --- | --- | --- | --- | --- | --- | --- | --- | --- | --- | --- | --- | --- |
| PKUSG-01 | Female | 32 | Left-sided | III | Adenocarcinoma | pMMR | Yes | XELOX | Yes | 9.50 | Dead | 19.50 |
| PKUSG-02 | Male | 35 | Left-sided | III | Adenocarcinoma | pMMR | Yes | XELOX | Yes | 18.38 | Dead | 23.18 |
| PKUSG-03 | Male | 39 | Left-sided | III | Adenocarcinoma | pMMR | Yes | XELOX | Yes | 15.39 | Dead | 45.76 |
| PKUSG-04 | Male | 33 | Left-sided | III | Adenocarcinoma | pMMR | Yes | XELOX | No | 65.00 | Alive | 65.00 |
| PKUSG-05 | Female | 37 | Left-sided | III | Adenocarcinoma | pMMR | Yes | XELOX | No | 47.93 | Alive | 47.93 |
| PKUSG-06 | Male | 53 | Left-sided | III | Adenocarcinoma | pMMR | Yes | XELOX | Yes | 14.66 | Dead | 31.27 |
| PKUSG-07 | Male | 51 | Left-sided | III | Adenocarcinoma | pMMR | Yes | XELOX | No | 54.21 | Alive | 54.21 |
| PKUSG-08 | Male | 54 | Left-sided | III | Adenocarcinoma | pMMR | Yes | XELOX | No | 62.10 | Alive | 62.10 |
| PKUSG-09 | Male | 57 | Left-sided | III | Adenocarcinoma | pMMR | Yes | XELOX | No | 55.92 | Alive | 55.92 |
| PKUSG-10 | Male | 58 | Right-sided | III | Adenocarcinoma | pMMR | Yes | XELOX | Yes | 1.97 | Dead | 5.92 |
| PKUSG-11 | Female | 61 | Left-sided | III | Adenocarcinoma | dMMR | Yes | XELOX | Yes | 16.08 | Dead | 31.20 |
| PKUSG-12 | Male | 60 | Left-sided | III | Adenocarcinoma | pMMR | Yes | XELOX | Yes | 7.53 | Dead | 18.84 |
| PKUSG-13 | Male | 65 | Left-sided | III | Adenocarcinoma | dMMR | Yes | XELOX | No | 54.41 | Alive | 54.41 |
| PKUSG-14 | Female | 65 | Right-sided | III | Adenocarcinoma | pMMR | Yes | XELOX | Yes | 11.51 | Dead | 21.34 |
| PKUSG-15 | Male | 72 | Left-sided | III | Adenocarcinoma | dMMR | Yes | XELOX | Yes | 61.68 | Alive | 65.46 |
| PKUSG-16 | Female | 73 | Right-sided | III | Mucinous adenocarcinoma | dMMR | Yes | CapeOX | Yes | 8.42 | Dead | 10.98 |
| PKUSG-17 | Male | 71 | Left-sided | III | Adenocarcinoma | pMMR | Yes | Capecitabine | No | 3.52 | Dead | 20.68 |
| PKUSG-18 | Female | 75 | Left-sided | III | Adenocarcinoma | dMMR | Yes | XELOX | No | 52.87 | Alive | 52.87 |
| PKUSG-19 | Male | 79 | Right-sided | III | Adenocarcinoma | pMMR | Yes | Capecitabine | Yes | 16.47 | Dead | 33.07 |
| PKUSG-20 | Male | 80 | Left-sided | III | Adenocarcinoma | pMMR | No |  | No | 1.41 | Dead | 1.41 |
| PKUSG-21 | Male | 28 | Left-sided | II | Adenocarcinoma | dMMR | Yes | XELOX | No | 69.17 | Alive | 69.17 |
| PKUSG-22 | Female | 30 | Right-sided | II | Adenocarcinoma | dMMR | No |  | No | 41.79 | Alive | 41.79 |
| PKUSG-23 | Female | 40 | Right-sided | II | Adenocarcinoma | dMMR | No |  | No | 64.77 | Alive | 64.77 |
| PKUSG-24 | Female | 49 | Right-sided | II | Mucinous adenocarcinoma | dMMR | No |  | No | 66.67 | Alive | 66.67 |
| PKUSG-25 | Male | 49 | Left-sided | II | Adenocarcinoma | dMMR | Yes | XELOX | No | 45.37 | Alive | 45.37 |
| PKUSG-26 | Male | 49 | Left-sided | II | Adenocarcinoma | dMMR | No |  | No | 41.33 | Alive | 41.33 |
| PKUSG-27 | Male | 52 | Left-sided | II | Adenocarcinoma | dMMR | No |  | No | 42.21 | Alive | 42.21 |
| PKUSG-28 | Male | 59 | Left-sided | II | Adenocarcinoma | dMMR | Yes | XELOX | No | 61.61 | Alive | 61.61 |
| PKUSG-29 | Male | 62 | Left-sided | II | Adenocarcinoma | dMMR | No |  | No | 36.85 | Alive | 36.85 |
| PKUSG-30 | Female | 64 | Right-sided | II | Adenocarcinoma | dMMR | No |  | No | 47.08 | Alive | 47.08 |
| PKUSG-31 | Female | 67 | Left-sided | II | Adenocarcinoma | dMMR | Yes | Capecitabine | No | 40.87 | Alive | 40.87 |
| PKUSG-32 | Male | 69 | Left-sided | II | Adenocarcinoma | dMMR | Yes | Capecitabine | No | 61.71 | Alive | 61.71 |
| PKUSG-33 | Male | 76 | Right-sided | II | Mucinous adenocarcinoma | dMMR | Yes | Capecitabine | No | 47.31 | Alive | 47.31 |
| PKUSG-34 | Male | 29 | Right-sided | II | Adenocarcinoma | pMMR | No |  | No | 65.46 | Alive | 65.46 |
| PKUSG-35 | Female | 58 | Right-sided | II | Adenocarcinoma | pMMR | No |  | No | 54.44 | Alive | 54.44 |
| PKUSG-36 | Female | 68 | Right-sided | II | Adenocarcinoma | pMMR | No |  | No | 65.39 | Alive | 65.39 |
| PKUSG-37 | Male | 76 | Right-sided | II | Adenocarcinoma | pMMR | No |  | Yes | 15.88 | Alive | 64.77 |
| PKUSG-38 | Male | 48 | Left-sided | III | Adenocarcinoma | pMMR | Yes | XELOX | No | 73.51 | Alive | 73.51 |
| PKUSG-39 | Male | 55 | Left-sided | II | Adenocarcinoma | pMMR | Yes | XELOX | Yes | 11.74 | Dead | 15.91 |
| PKUSG-40 | Female | 58 | Left-sided | I | Adenocarcinoma | pMMR | No |  | No | 71.21 | Alive | 71.21 |
| PKUSG-41 | Male | 58 | Left-sided | II | Adenocarcinoma | pMMR | Yes | XELOX | Yes | 2.93 | Alive | 62.24 |
| PKUSG-42 | Male | 59 | Left-sided | I | Adenocarcinoma | pMMR | No |  | Yes | 58.32 | Alive | 72.62 |
| PKUSG-43 | Male | 59 | Left-sided | III | Adenocarcinoma | pMMR | Yes | XELOX | No | 74.89 | Alive | 74.89 |
| PKUSG-44 | Female | 61 | Left-sided | II | Adenocarcinoma | pMMR | No |  | No | 43.59 | Alive | 43.59 |
| PKUSG-45 | Male | 61 | Left-sided | II | Adenocarcinoma | pMMR | Yes | XELOX | Yes | 10.92 | Dead | 55.69 |
| PKUSG-46 | Male | 62 | Left-sided | II | Adenocarcinoma | pMMR | No |  | No | 44.52 | Alive | 44.52 |
| PKUSG-47 | Male | 63 | Left-sided | II | Adenocarcinoma | pMMR | No |  | No | 41.16 | Alive | 41.16 |
| PKUSG-48 | Female | 63 | Left-sided | II | Adenocarcinoma | pMMR | Yes | XELOX | No | 58.32 | Alive | 58.32 |
| PKUSG-49 | Female | 65 | Left-sided | III | Adenocarcinoma | pMMR | No |  | Yes | 57.86 | Dead | 67.79 |
| PKUSG-50 | Male | 63 | Left-sided | II | Adenocarcinoma | pMMR | No |  | Yes | 12.39 | Alive | 65.69 |
| PKUSG-51 | Male | 67 | Left-sided | II | Adenocarcinoma | pMMR | No |  | Yes | 41.36 | Dead | 52.34 |
| PKUSG-52 | Female | 69 | Left-sided | I | Adenocarcinoma | pMMR | No |  | No | 69.37 | Alive | 69.37 |
| PKUSG-53 | Male | 84 | Right-sided | III | Mucinous adenocarcinoma | pMMR | No |  | Yes | 8.58 | Dead | 35.61 |
| PKUSG-54 | Male | 80 | Right-sided | I | Adenocarcinoma | pMMR | No |  | No | 72.62 | Alive | 72.62 |
| PKUSG-55 | Male | 69 | Right-sided | II | Adenocarcinoma | pMMR | Yes | XELOX | No | 66.18 | Alive | 66.18 |
| PKUSG-56 | Male | 62 | Right-sided | II | Adenocarcinoma | dMMR | No |  | No | 78.38 | Alive | 78.38 |
| PKUSG-57 | Female | 69 | Right-sided | III | Adenocarcinoma | pMMR | Yes | XELOX | No | 50.07 | Alive | 50.07 |
| PKUSG-58 | Male | 81 | Right-sided | II | Adenocarcinoma | pMMR | Yes | Capecitabine | No | 15.88 | Dead | 15.88 |
| PKUSG-59 | Female | 60 | Right-sided | II | Mucinous adenocarcinoma | NA | No |  | No | 83.67 | Alive | 83.67 |
| PKUSG-60 | Female | 48 | Right-sided | NA | NA | pMMR | No |  | No | 45.93 | Alive | 45.93 |
| PKUSG-61 | Male | 64 | Right-sided | II | Adenocarcinoma | pMMR | Yes | Capecitabine | No | 73.78 | Alive | 73.78 |
| PKUSG-62 | Male | 62 | Right-sided | III | Mucinous adenocarcinoma | dMMR | Yes | XELOX | No | 82.98 | Alive | 82.98 |
| PKUSG-63 | Female | 69 | Right-sided | II | Adenocarcinoma | pMMR | Yes | Capecitabine | No | 50.01 | Alive | 50.01 |
| PKUSG-64 | Male | 74 | Right-sided | II | Adenocarcinoma | pMMR | No |  | No | 51.42 | Alive | 51.42 |
| PKUSG-65 | Male | 84 | Right-sided | I | Adenocarcinoma | dMMR | No |  | No | 42.15 | Alive | 42.15 |
| PKUSG-66 | Male | 81 | Right-sided | II | Adenocarcinoma | pMMR | No |  | No | 41.06 | Dead | 41.06 |
| PKUSG-67 | Male | 42 | Right-sided | III | Adenocarcinoma | pMMR | Yes | XELOX | No | 48.23 | Alive | 48.23 |
| PKUSG-68 | Male | 55 | Right-sided | II | Mucinous adenocarcinoma | pMMR | Yes | XELOX | No | 51.12 | Alive | 51.12 |
| PKUSG-69 | Male | 48 | Right-sided | I | Adenocarcinoma | pMMR | No |  | Yes | 6.67 | Dead | 17.52 |
| PKUSG-70 | Female | 70 | Right-sided | III | Adenocarcinoma | pMMR | Yes | XELOX | No | 75.19 | Alive | 75.19 |
| PKUSG-71 | Male | 83 | Right-sided | III | Adenocarcinoma | dMMR | No |  | Yes | 5.06 | Dead | 15.25 |
| PKUSG-72 | Female | 81 | Right-sided | II | Adenocarcinoma | pMMR | No |  | No | 68.45 | Alive | 68.45 |
| PKUSG-73 | Female | 76 | Right-sided | II | Adenocarcinoma | dMMR | No |  | No | 80.68 | Alive | 80.68 |
| PKUSG-74 | Female | 73 | Right-sided | I | Adenocarcinoma | pMMR | No |  | No | 49.84 | Alive | 49.84 |
| PKUSG-75 | Female | 76 | Right-sided | III | Adenocarcinoma | pMMR | No |  | No | 0.85 | Dead | 0.85 |
| PKUSG-76 | Female | 62 | Right-sided | I | Adenocarcinoma | pMMR | No |  | No | 82.22 | Alive | 82.22 |
| PKUSG-77 | Male | 33 | Right-sided | II | Adenocarcinoma | pMMR | Yes | Capecitabine | No | 58.16 | Alive | 58.16 |
| PKUSG-78 | Male | 74 | Right-sided | III | Adenocarcinoma | pMMR | Yes | Capecitabine | No | 60.23 | Alive | 60.23 |
| PKUSG-79 | Male | 25 | Right-sided | III | Adenocarcinoma | pMMR | Yes | XELOX | No | 49.84 | Alive | 49.84 |
| PKUSG-80 | Female | 36 | Right-sided | III | Adenocarcinoma | pMMR | Yes | XELOX | No | 49.41 | Alive | 49.41 |
